# Direct Integration of Ionic Liquid Gel Sensors onto Microfibrous Face Mask Substrates for Wearable Respiratory Health Monitoring

**DOI:** 10.1021/acsabm.5c01939

**Published:** 2026-02-17

**Authors:** Ziqi Qing, Seokmin Choi, Matthew S. Brown, Md Abid Hasan Shanto, Yincheng Jin, Ahyeon Koh, Zhanpeng Jin, Jeffrey M. Mativetsky

**Affiliations:** † Materials Science and Engineering, 14787 Binghamton University, Binghamton, New York 13902, United States; ‡ Department of Computer Science and Engineering, 12292 University at Buffalo, Buffalo, New York 14260, United States; § Department of Biomedical Engineering, Binghamton University, Binghamton, New York 13902, United States; ∥ School of Computing, Binghamton University, Binghamton, New York 13902, United States; ⊥ Department of Physics, Applied Physics and Astronomy, Binghamton University, Binghamton, New York 13902, United States

**Keywords:** wearable electronics, ionic liquid gel, microfibrous substrate, humidity sensing, respiratory health monitoring

## Abstract

Mask-integrated respiratory sensors are promising for noninvasive monitoring of respiratory health outside of clinical settings, for example, to support at-home patient monitoring and telemedicine. Studies have shown the feasibility of attaching flexible and off-the-shelf sensors to face masks; however, the potential benefits of incorporating sensor materials directly onto the breathable microfibrous mesh of face masks have hardly been explored. In this work, we integrate respiratory sensors on flat polyethylene terephthalate substrates and microfibrous polypropylene face masks using an ionic liquid gel (ILG) to detect changes in local humidity during breathing. Under a DC voltage, the ILG sensors exhibit a superlinear dependence of current on humidity, resulting in enhanced sensitivity at the high humidity levels found in exhaled breath. By varying the composition of the ILG, we find an inverse relationship between the sensing signal strength and sensing kinetics, offering insights into the sensing mechanisms. Freestanding ILG films are found to be resilient under mechanical strain with humidity sensing still possible at 120% strain. Devices that are scalably integrated onto face masks produce sensors that are four times less sensitive to extreme bending at an angle of 150°. Preliminary artificial intelligence (AI) analysis of breathing patterns identifies coughs that are interspersed with regular breathing with 91% accuracy, showing the potential of AI-supported wearable masks for autonomous symptom tracking.

## Graphical abstract

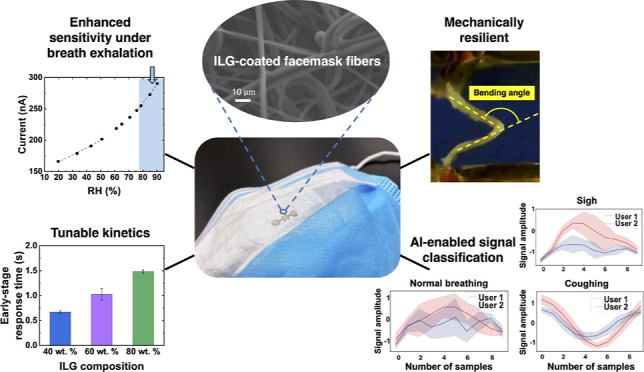

## Introduction

Although chronic lower respiratory disease (CLRD), including chronic obstructive pulmonary disease (COPD) and asthma, is the sixth leading cause of death in the United States,^1^ most cases of CLRD remain undiagnosed.^2,3^ Given the critical impact of early detection and treatment on health outcomes,^4^ there is a need for tools that facilitate access to respiratory health data. Wearable respiratory sensors^5–12^ open the possibility of continuously and noninvasively observing breathing patterns for at-home patient monitoring, telemedicine, and clinical studies of respiratory disease and treatments. Coupling wearable electronics with AI-powered symptom tracking and disease detection creates further opportunities for addressing reduced respiratory health outcomes in rural and disadvantaged communities that have limited health infrastructure.^13^

During exhalation, humid air from the lungs exits the nose or mouth, with a relative humidity (RH) that is close to saturation,^14–16^ making humidity a sensitive marker of breathing activity. Many materials have been reported as humidity-sensing materials, including carbon-based nanomaterials,^17–19^ metal oxides,^20,21^ paper,^22^ and polymers.^23,24^ Ionic liquid gels (ILGs) are emerging as promising candidates for wearable sensing owing to their low and tunable Young’s modulus of 3 ∼ 1000 MPa^25^ that approaches that of the skin, while maintaining ionic conductivity and sensing properties.^26,27^ ILGs incorporate ionic liquids (ILs) into a polymer network^28,29^ through weak intermolecular interactions, such as hydrogen bonds.^28^ The ionic conductivity of ILGs is derived from the incorporation of ILs, which are molten salts at room temperature^30^ that exhibit good thermal stability and a wide electrochemical window.^31,32^

Initial studies have shown that ILGs can be used as bend-resilient humidity^33^ and respiratory sensors.^34–37^ A hyperbranched dendrite-like polymer backbone was used in one study, along with various counter-anions to tune the hydrophobicity of the resulting ILG and improve the linear impedance response to humidity.^37^ Another study used bar printing to produce a capacitive ILG sensor that showed a linear response to humidity over a broad humidity range and demonstrated wireless communication in a wrist-wearable form factor.^33^ In another case, electrospun ILG fiber networks, which present a large surface-to-volume ratio for water adsorption and desorption, exhibited a high sensitivity,^34,35^ while responding to periodic humidity changes as fast as 120 Hz.^35^ In a different study, multifunctional sensing in electrospun ILG fiber networks was demonstrated with limited crosstalk, to detect humidity, via the electrical resistance, and pressure, via the capacitance.^34^ Mask attachment enabled differentiation between nasal and mouth breathing. Further study is needed, however, to elucidate structure–function relationships, performance limits, and sensing mechanisms in ILG humidity and respiratory sensors.

Mask-integrated respiratory sensors have so far made use of sensors built on planar substrates that are mounted onto commercial face masks.^38–40^ As an alternative, direct coating of sensing materials onto microfibrous face masks presents an opportunity to imbue sensing materials with a microfibrous morphology that can improve the conformal properties of wearable respiratory sensors, increase the specific area of the sensing material, maintain substrate breathability, and simplify fabrication by reducing the number of fabrication steps and device layers. A recent study attributed the enhanced ammonia sensing performance of polyaniline/graphene-functionalized face mask substrates to a large specific surface area for gas adsorption and the availability of continuous conduction pathways for charge carriers.^41^ Despite the relevance of face masks for wearable respiratory monitoring, composites composed of microfibrous mask substrates and humidity-sensing materials have not yet been considered.

This study incorporates an ILG and printed electrodes directly onto microfibrous mask substrates to enable wearable respiratory health monitoring. Unlike previous studies that coat sensing materials onto planar substrates and attach the substrates to a face mask, we show that not only is device integration onto porous face mask substrates possible but it also brings superior signal stability under bending. By studying free-standing ILG films, we gain insights into the origin of an opposite response to bending on planar and microfibrous mask substrates. By varying the ILG composition, we gain insights into the respiratory sensing mechanism and tunable sensing kinetics. Realtime breath sensing is made possible by a rapid response to humidity during breathing along with an increased sensitivity at the high humidities present during exhalation. Finally, machine learning was shown to successfully classify breathing activities such as coughing, showing the potential for telehealth and at-home patient health monitoring.

## Results and Discussion

As shown in Figure 1a, the ILG is composed of ionic liquid 1-ethyl-3-methylimidazolium bis­(trifluoromethylsulfonyl)­imide ([EMIM]­[TFSI]) and copolymer poly­(vinylidene fluoride-*co*-hexafluoropropylene) P­(VDF-HFP), where the IL is incorporated into the copolymer through the ion–dipole interaction between the IL’s cation and the copolymer P­(VDF-HFP).^42,43^ Fourier transform infrared spectroscopy (FTIR) spectra show that the ILG mainly contains peaks from the IL, with peaks at 879 cm^–1^ and 1405 cm^–1^ confirming the presence of the copolymer in the ILG (Supporting Information, Figure S1). According to available material safety data, the copolymer is nonhazardous and the IL has a low oral and dermal acute toxicity but can cause eye damage and skin irritation during direct contact. To ensure that the respiratory sensor does not come into contact with the user, the mask-integrated sensor is sandwiched between uncoated mask layers.

**Figure 1:**
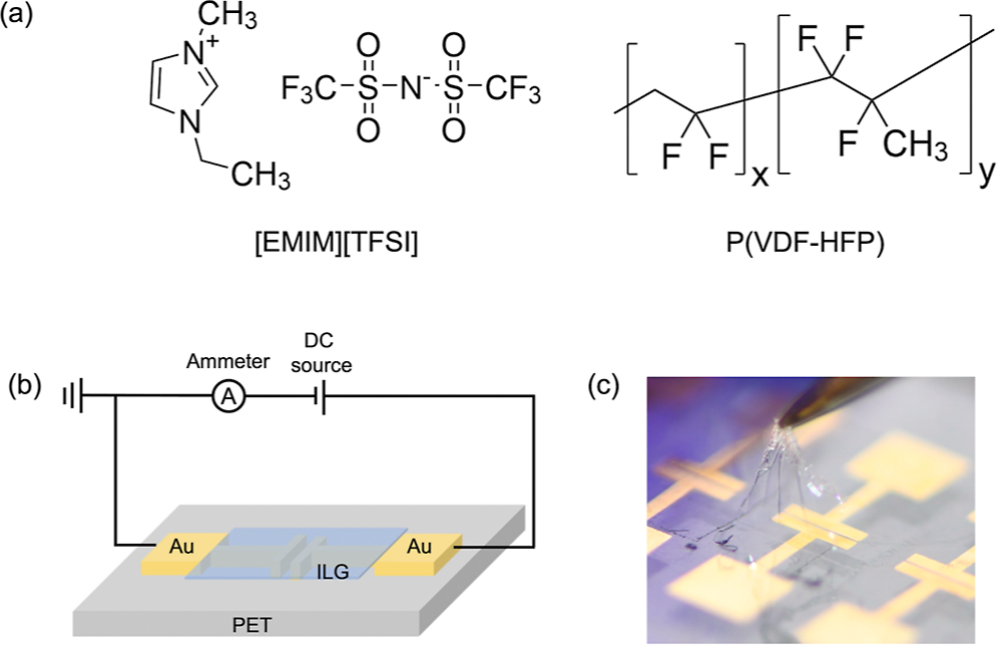
(a) Molecular structure of IL [EMIM]­[TFSI] and copolymer P­(VDF-HFP). (b) Schematic of the sensor structure and the setup for electrical measurement. (c) Photo of a flexible ILG film being pulled from a device.

Before integrating ILG sensors onto mask substrates, we first measured the performance on flat polyethylene terephthalate (PET) substrates to serve as a reference point and to gain insight into the role of ILG composition. The ILG sensor structure for devices on PET and the setup for electrical measurement are shown in Figure 1b. ILG films were spin-coated onto flexible PET substrates with prepatterned gold electrode pairs. Humidity sensing was performed by applying 2 V across an electrode pair, while measuring the current. As demonstrated in Figure 1c, the ILG is highly flexible, making it promising for flexible electronics and wearable applications.

To investigate how the ILG composition affects the sensor’s response to humidity, three ILG compositions were considered. Figure 2a shows the current as a function of RH for the ILG containing 40, 60, and 80 wt % IL. For all three devices, the current increases as the humidity increases, with a steeper slope at higher RH and for higher wt % IL. The sensitivity in the low and high RH regime was extracted from the slope Δ*I*/ΔRH, where Δ*I* is the current change and ΔRH is the humidity change. As shown in Figure 2b,c, the sensitivity increases with the IL content in both the low RH and high RH regimes. Furthermore, for all compositions, the sensitivity in the high RH regime (Figure 2c) is significantly higher than the sensitivity in the low RH regime (Figure 2b). The greatest sensitivity increase is seen for 80 wt % IL, with a high RH sensitivity that is 30.5 times greater than the sensitivity at low RH. The elevated sensitivity at high humidity makes this sensor well suited for detecting breathing, which results in an RH in the range of 66–98% during exhalation.^14–16^ It should be noted that IL content below 40 wt % and above 80 wt % were not considered in this analysis since lower IL contents led to poor device sensitivity, while higher IL contents led to the leakage of the IL as ion–dipole interactions with the P­(VDF-HFP) copolymer became saturated.

**Figure 2:**
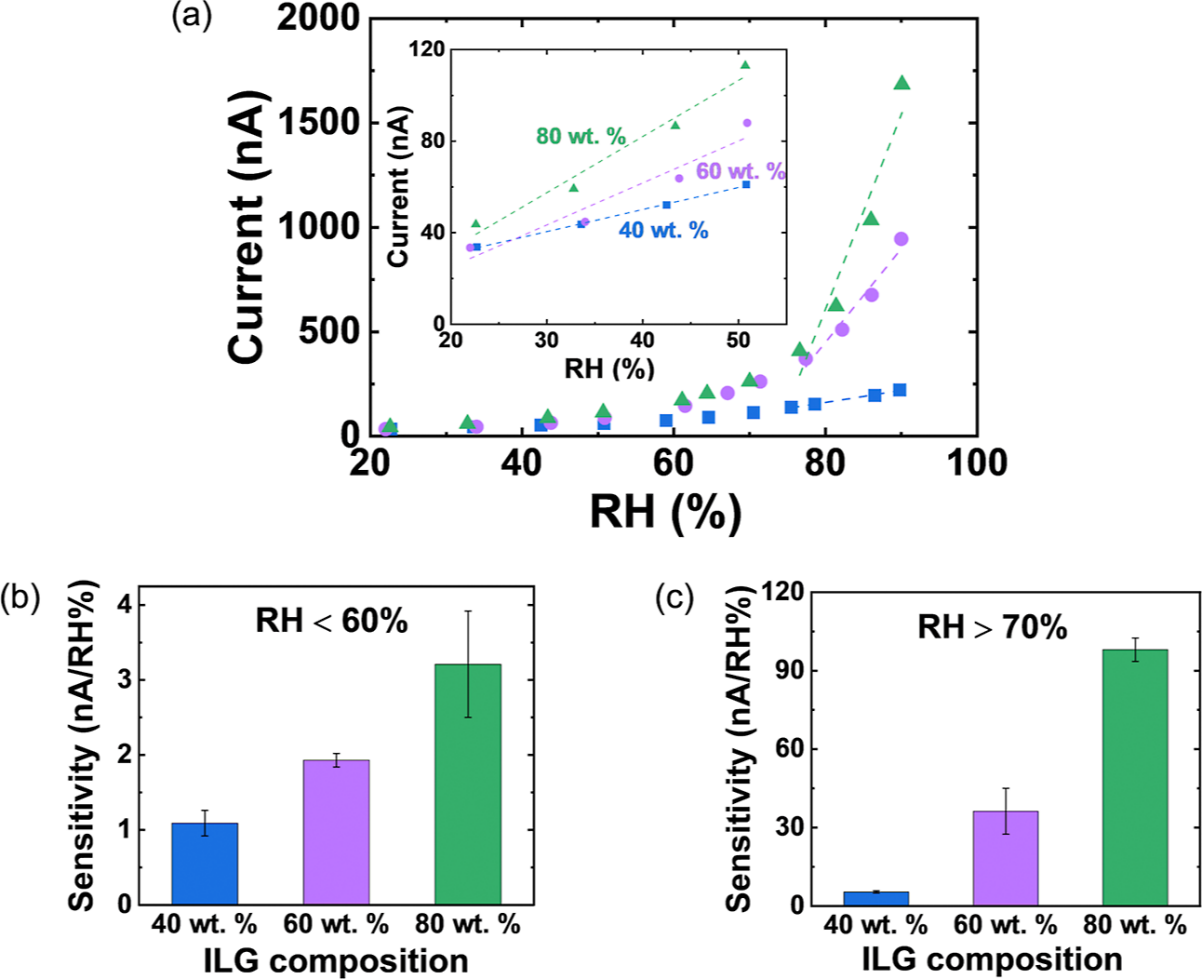
(a) Current as a function of humidity for three compositions of the ILG, with linear fits in the low humidity and high humidity regimes. The inset shows an expanded view of the low humidity regime. Sensitivity for the three ILG compositions in (b) the low humidity and (c) high humidity regime.

It should be noted that although the film thickness varies with ILG composition (1.7 μm, 2.8 μm, and 5.0 μm for the 40, 60, and 80 wt % ILG, respectively), varying the film thickness from 1.6 to 6.7 μm while keeping the composition fixed at 60 wt % did not result in appreciable changes in the current response (Supporting Information, Figure S2). Film thickness therefore did not play a role in the observed composition dependence. The lack of dependence on film thickness also suggests that water at or near the film’s surface dominates the device response, rather than absorption into the bulk.

To examine the sensing kinetics, we measured the current response to rapid switching between 11% and 75% RH (Figure 3a). Here, the current change is defined as Δ*I* = *I*–*I*_0_, where *I* is the current and *I*_0_ is the average initial current at 11% RH. In each case, the current rapidly increases, in a time scale of about 1 s, then approaches saturation at a slower rate, in a time scale of minutes. In accordance with the sensitivity measurements (Figure 2), the current change is more pronounced for higher IL contents.

**Figure 3:**
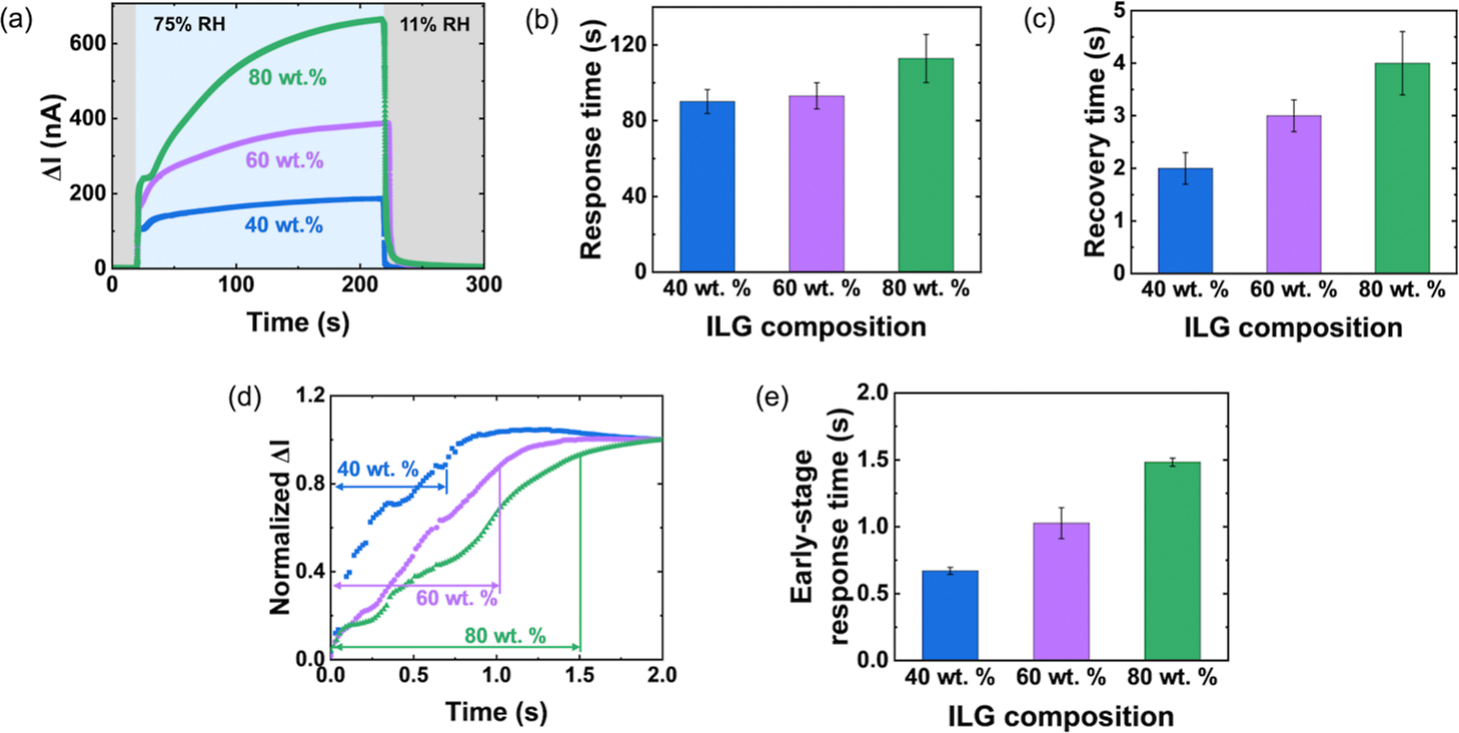
(a) Current change during rapid switching between 11% and 75% RH, (b) response time, and (c) recovery time for three sample compositions. (d) Early-stage response curves, normalized to the current change at 2 s and (e) early stage response times.

To evaluate the response and recovery times, we determined the time required to reach 90% of the current change over a defined time period. The long-term response was determined over a time of 200 s, while the early-stage response was extracted over the first 2 s. As shown in Figure 3b, the long-term response time increases with IL content, from 90 s at 40% IL to 113 s at 80% IL. A similar trend is observed for the recovery times (Figure 3c), though the recovery is quicker, ranging from 2 s at 40% IL to 4 s at 80% IL. Figure 3d shows the early-stage response region and the corresponding response times (Figure 3e), which are as fast as 0.7 s at 40% IL and 1.5 s at 80% IL. It can be concluded that the long-term response, early-stage response, and recovery time all increase as the IL content increases.

In the context of respiratory sensing, the normal respiratory rate (RR) is 12–20 breaths per minute (bpm) for adults.^44^ One normal breath therefore takes 3–5 s, with about 1.5–2.5 s for inhalation and a similar time for exhalation. The sensor’s early-stage response and recovery times, particularly at low IL content, are comparable to the time for normal inhalation and exhalation, ensuring that the sensor can respond rapidly enough to track breathing. On the other hand, the sensor’s sensitivity (Figure 2) is reduced at low IL content. To balance the need for a sensitive and rapid response, we selected the 60 wt % IL composition for subsequent studies. It should be noted that, in practice, the device response is much quicker than the early-stage response and recovery times since breath detection does not require that the signal strength reaches as high as the 90% criterion used to quantify the kinetics.

To understand the origin of the inverse relationship between sensitivity and kinetics as the IL content is varied, we consider the interactions between the ILG and water. At the initial stage of water adsorption, water molecules are immobilized on the IL through ionic-hydrogen bonding,^45^ with the oxygen atoms from the anion [TFSI] being the dominant bonding site for hydrogen atoms from water.^46^ Subsequent water layers can be adsorbed through hydrogen bonding between water molecules. In these upper layers, water dissociation can generate protons that diffuse under an applied voltage, generating a humidity-dependent current. At low humidity levels, limited water buildup will limit the proton current. Similarly, an ILG with a low IL content presents fewer bonding sites for water molecules, thus reducing the water adsorption and proton current.

The composition dependence of the sensor data is consistent with the proposed scheme. First, as shown in Supporting Information, Figure S3, the water contact angle decreases as the IL content increases, from 86° at 40 wt % IL to 56° at 80 wt %, reflecting an increased hydrophilicity of the ILG surface as the IL content is increased. Second, the roughness of the ILG increases with IL content, from 35 nm for 40 wt % IL to 119 nm for 80 wt % IL, measured over a 100 μm^2^ area (Supporting Information, Figure S4). This rise in roughness correspondingly enlarges the effective surface area from 103 to 136 μm^2^. The increased area for water adsorption for higher IL contents in turn results in increased proton generation and current flow under an applied voltage. Consequently, devices with the highest IL content (80 wt % IL) exhibit the highest sensitivity. On the other hand, devices with a high IL content lead to longer response and recovery times. This can be because of the longer time needed to adsorb and desorb a more extensive water layer.

It should be noted that at 0% RH, the device current is due to the ionic conductivity of the IL. As shown in Supporting Information, Figure S5, the ionic current at 0% RH increases with increasing IL content. For the 80 wt % ILG, the current at 75% RH is almost 19 times higher than it is at 0% RH, meaning that the intrinsic ionic current is small compared to the humidity-induced current.

As shown in Supporting Information, Figure S6, the 60 wt % ILG sensor exhibits a stable response to breathing, with no signal degradation after several breathing tests recorded over a 24 h period. This period is longer than the typical usage life of a disposable mask. For practical field implementation, a single use mask-integrated sensor could be paired with a reusable wireless module. Further tests showed that after storage in nitrogen for 4 months, the respiratory sensing performance was indistinguishable (Supporting Information, Figure S7). The sensor responds reproducibly to rapid humidity changes (Figure S8) and exhibits a hysteresis error of 6.6% during sensitivity tests (Figure S9). To monitor the effect of temperature on sensor performance, we tracked the average peak current during breathing (the on-current) while a 60 wt % ILG sensor was held between room temperature and 40 °C. As shown in Supporting Information, Figure S10, the on-current decreases as the temperature increases, which is expected since water will evaporate more readily from the ILG surface at elevated temperatures. Up to 30 °C, the current changes are minimal, under 14%, but become more appreciable, above 49% at 35 °C and higher. This result shows that the sensors are appropriate for use in typical indoor conditions and potentially hot outdoor conditions, though with a reduced signal amplitude. In usage cases where the absolute signal strength is not critical, such as extracting a respiratory rate and breathing waveform, the sensor can be used over a broad temperature range.

To evaluate the resilience of the ILG’s humidity sensing properties under mechanical strain, we performed sensing tests on stretched free-standing ILG films (Figure 4a). As shown in the stress–strain curve in Figure 4b, the ILG film can be elongated to 140% strain before it fractures. At strains even as high as 120%, a distinct response to humidity changes is still evident. Figure 4c shows the current response of an ILG film under 100% strain during rapid switching between 37% and 60% RH environments. As seen in Figure 4d, the average peak current at 60% RH (*I*_60_) and the baseline current at 37% RH (*I*_37_) gradually decrease with increasing strain. This can be explained, in part, by an increase in resistance caused by the increased distance for charge transport during stretching, since the electrical resistance is given by *R* = ρ*L*/*A*, where ρ is the film’s resistivity, *L* is the length, and *A* is the cross-sectional area. The increase in transport length during stretching will affect both the resistance associated with the flow of protons from water dissociation and ions in the IL. In addition, the cross-sectional area of the ILG will decrease, and the density of hydrogen bonding sites for water adsorption will be reduced upon stretching, leading to further resistance increases. Figure 4e shows the percent current change, (*I*_60_–*I*_37_)/*I*_37_ × 100%, which decreases from 54% to 27% over the range of 0% to 120% strain. This result reveals that an appreciable sensing signal is retained even under extreme strain.

**Figure 4:**
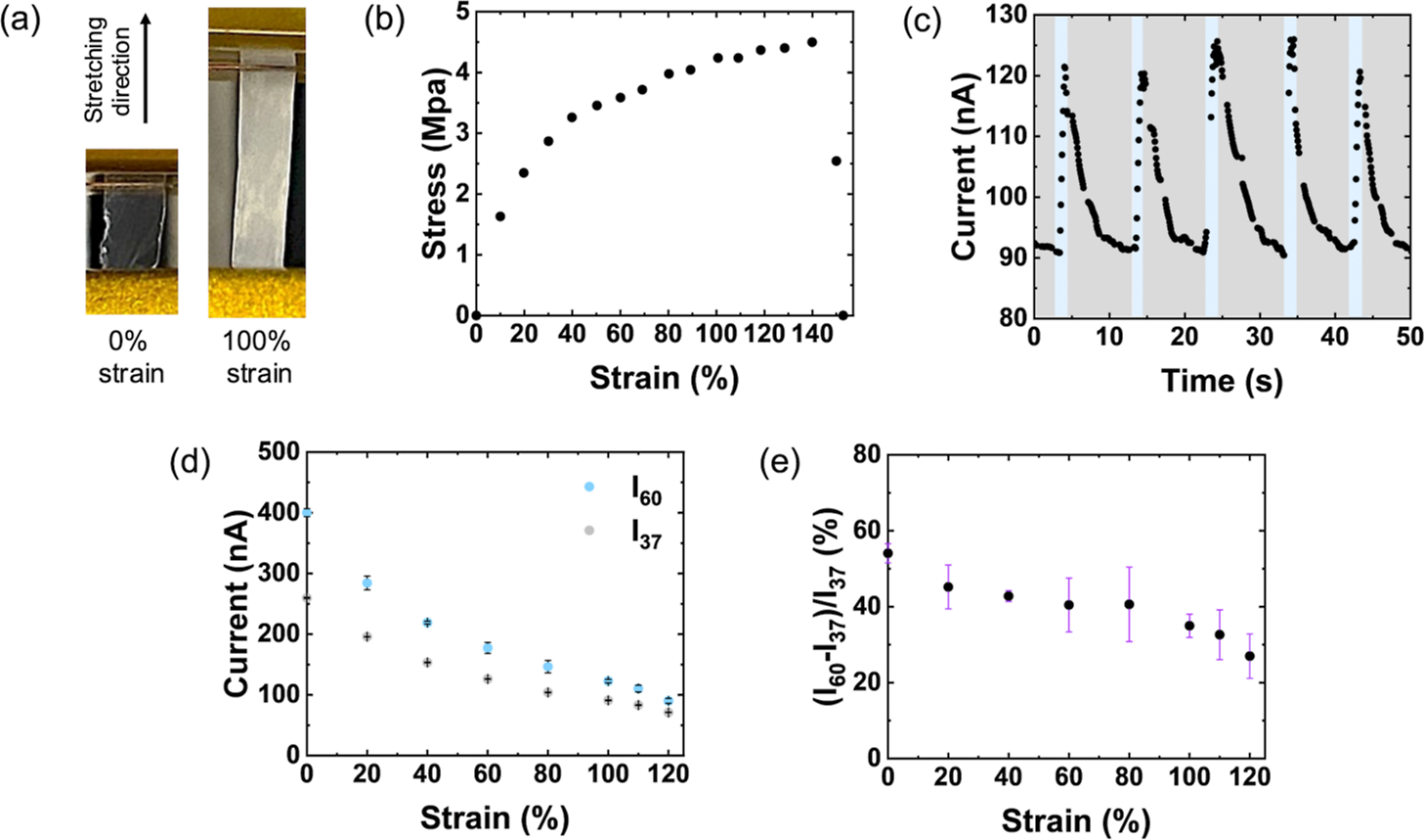
(a) Photographs of a 2.8 μm thick free-standing ILG film with 60 wt % ILG being stretched along the labeled direction to 100% strain and (b) a stress–strain curve. (c) The response to rapid humidity changes at 100% strain. The blue shading represents exposure to 60% RH. The gray blocks represent exposure to 37% RH. (d) Baseline current at 37% RH and peak current at 60% RH, and (e) percent current change as a function of strain.

To explore the potential for integrating ILG humidity sensors directly onto microfibrous mask substrates for wearable health sensing, we used a simple and scalable approach (illustrated in Supporting Information, Figure S11). Silver/silver chloride electrodes were patterned onto polypropylene mask layers by using screen printing. The silver/silver chloride ink’s viscosity of 40,000 ± 10,000 cP allows the ink to bridge the microfibrous features of the middle filter layer of a disposable polypropylene mask during printing. Patterning fidelity was reduced, however, when printing electrodes onto the outer mask layer (Supporting Information, Figure S12a) because of its coarser and more porous morphology. As shown in Supporting Information, Figure S12b, the mask’s outer layer presents a sparse and porous network of microfibers with diameters of 29 ± 2 μm, while the middle filter layer exhibits a dense mesh of smaller (3 ± 2 μm diameter) microfibers that better support the electrode coating.

Following electrode printing, a droplet of dilute ILG solution was applied to the mask to form a thin conformal coating throughout the substrate’s three-dimensional fibrous network. ILG dilution allows for further tuning of both the sensing signal strength and kinetics (Supporting Information, Figure S13). By diluting the ILG solution by a factor of 4, the initial response and recovery times were reduced from half a minute to a few seconds. The current level, on the other hand, decreased upon dilution. These effects could result from less extensive ILG coverage on the mask’s microfibrous network leading to less water adsorption.

A mask-integrated ILG device with the ILG diluted by a factor of 4 is shown in Figure 5a, while Figure 5b shows the microstructure of an uncoated and ILG-coated mask. It can be seen that the ILG coating does not alter the fibrous mask morphology. Top-view (Figure 5c) and cross-sectional (Figure 5d) energy-dispersive spectroscopy (EDS) maps of the fluorine from the ILG reveal that the ILG coats the fibers throughout the entire thickness of the mask. The conformal coating serves to maximize the sensing area of the device while maintaining the mask’s porosity. The intimate wrapping of the ILG around the polypropylene fiber network also creates a robust attachment that cannot be peeled from the surface like the ILG film on PET (Figure 1c).

**Figure 5:**
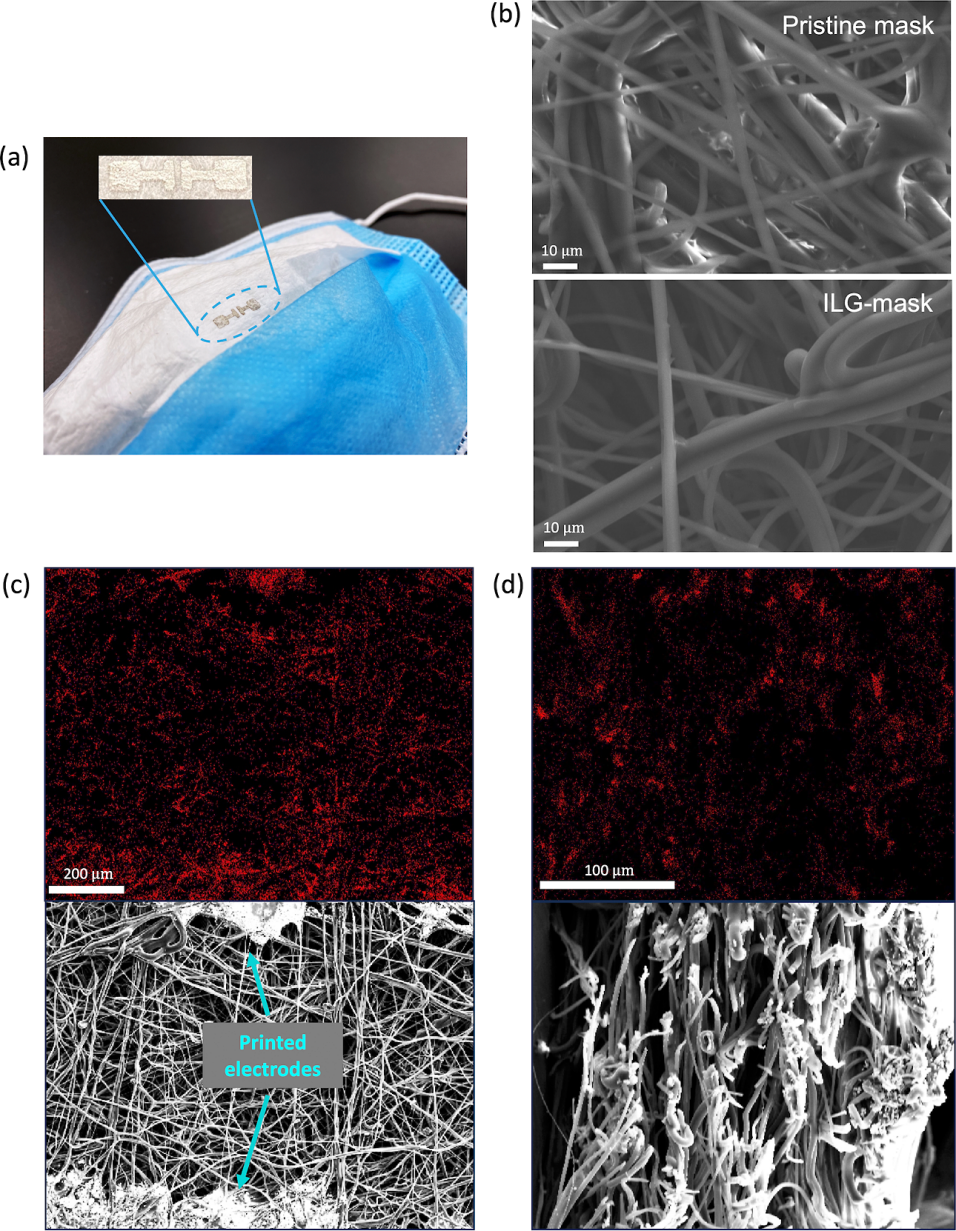
(a) Photo and close-up view of a respiratory sensor fabricated directly on a face mask. (b) Scanning electron microscopy (SEM) image of a mask’s filter layer, without and with an ILG-coating. (c) Energy-dispersive X-ray spectroscopy (EDS) map of the fluorine present in an ILG-coated mask and the corresponding SEM image. (d) EDS fluorine map and SEM image of an ILG-coated mask’s cross-section.

Similar to devices prepared on PET substrates, the mask-integrated ILG devices feature an increase in current with increasing RH, with a steeper increase above 70% RH (Figure 6a). In the low RH regime (RH < 60%), the ILG-mask device has a sensitivity of 1.1 nA/RH %, while in the high RH regime (RH > 70%), the sensitivity rises to 2.9 nA/RH %. As shown in Supporting Information, Figure S14, the adsorption and desorption curves exhibited a minor hysteresis error of 3.0%. Like the devices prepared on PET, mask-integrated devices also display a fast initial response and a slower subsequent response when the humidity is rapidly switched from 11% to 75% RH (Figure 6b). The initial response and recovery time are 1.1 ± 0.1 and 3.6 ± 0.6 s, respectively, again comparable to the time scale for breathing.

**Figure 6:**
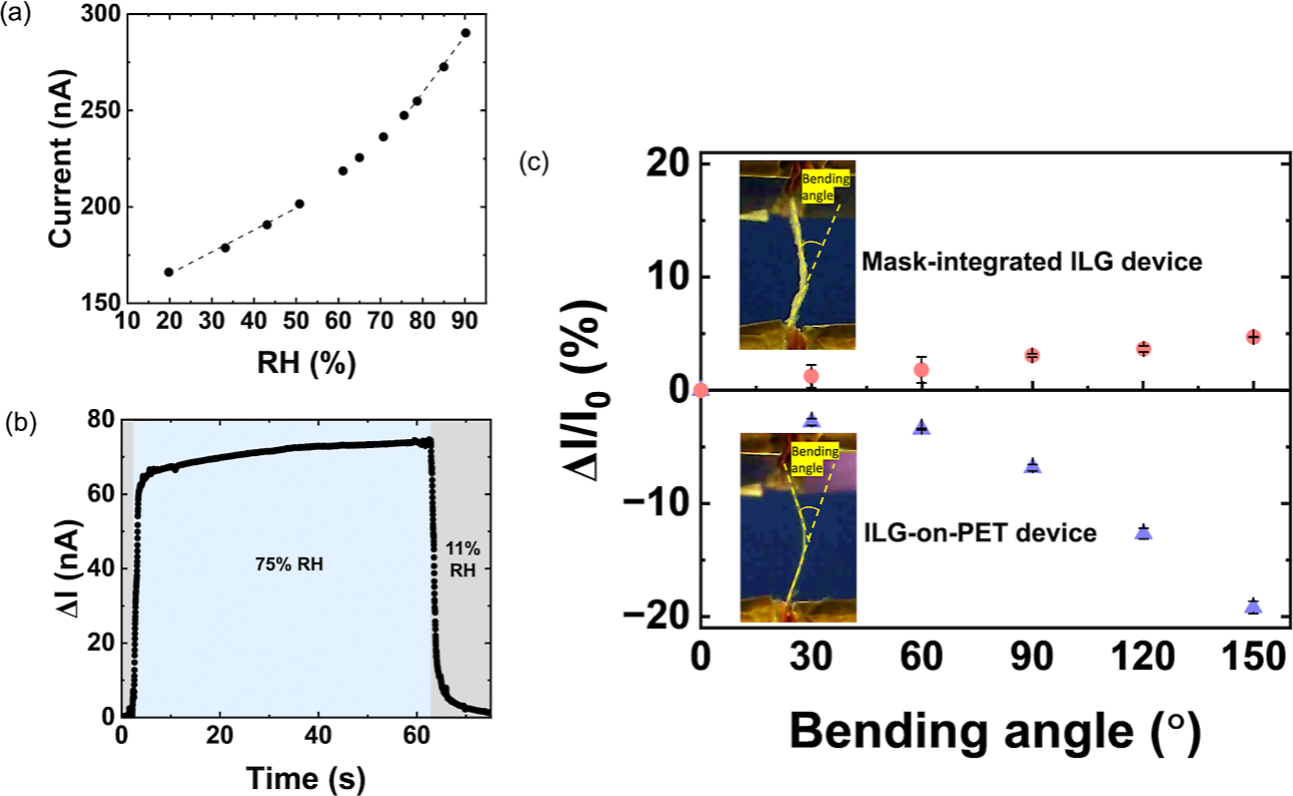
(a) Current as a function of humidity for a mask-integrated ILG device (60 wt % IL), with linear fits in the low and high RH regimes. (b) Response kinetics during rapid switching between 11% and 75% RH. (c) Comparison of the effect of bending on current for a mask-integrated device and a device on PET at 60% RH. The inset images show a side view of the devices during bending and the defined bending angles.

In contrast, however, the mask-integrated devices respond differently to bending than the devices on PET. Figure 6c shows the percent current change as a function of the bending angle for a mask-integrated ILG device and a device on PET (ILG-on-PET device), with the top surface of the device under tension (bending outward). The ILG-on-PET devices were prepared by using the same procedure for screen printing the electrodes. Interestingly, the mask-integrated ILG device shows a positive change in current with a bend angle, while the ILG-on-PET device exhibits a negative change in current. In addition, the ILG-on-PET device has a larger percent current change than the mask-integrated ILG device at each bending angle, especially at bending angles greater than 90°. At a bending angle of 150°, the ILG-on-PET device has a current change of −19.2 ± 0.6%, while the mask-integrated ILG device has a much smaller current change of 4.7%. This result shows that the mask-integrated ILG device is less sensitive to bending and is only impacted by a small amount, even at extreme bending angles.

To decouple the role of the ILG and electrodes, we performed bending measurements on continuous screen-printed electrodes prepared on mask and PET substrates (Supporting Information, Figure S15). Both samples exhibit a negative change in current, with the PET substrate again leading to a larger current change, up to −20.6% at 150°. Under the same bending conditions, the mask substrate shows a current change of −9.8% at 150°. In the device circuit, however, the electrodes only contribute a small portion of the series resistance because the electrode resistance during bending remains in the range of a few Ohms while the device resistance reaches as much as 10^7^ Ω, indicating that the electrodes are not a significant source of resistance during bending.

The divergent sign for the current change while bending the mask-integrated ILG and ILG-on-PET devices can be explained by the difference in the ILG geometry in the two cases. For the ILG-on-PET device, the ILG is under tension, and the surface of the ILG is stretched, thus reducing the current level, as seen in the strain experiments on free-standing films (Figure 4). On the other hand, in the mask-integrated ILG device, according to the EDS data (Figure 5c,d), the ILG coats the fibrous polypropylene network throughout the depth of the mask layer. If the fibers are coated around their entire circumference, then the bending of the mask will lead to tension at one side of the fibers and compression at the opposite side. Under compression, proton transport can be promoted by increasing the density of hydrogen bonding sites for water adsorption and decreasing the proton transport path length. Based on the increasing current trend during bending, the side of the fibers that is under compression has a greater impact on the current than the side that is under tension due to the current favoring the path of least resistance. A similar trend was observed in the case of a free-standing ILG film, which also has a surface under tension and a surface under compression during bending (Supporting Information, Figure S16).

The reduced sensitivity to bending for the mask-integrated device versus the ILG-on-PET device can be explained by differences in the magnitude of the surface bending strain. The surface bending strain (*S*) can be estimated from the equation *S* = *h*/2*R*, where *h* is the thickness of the substrate and *R* is the bending radius of the device.^47^ The PET substrate has a thickness of 99 ± 4 μm, while the fibers of the mask’s filter layer are only 3 ± 2 μm in diameter. According to the above equation, under the same bending conditions, the surface bending strain of the mask-integrated ILG is 33 times less than the ILG on PET, highlighting the mechanical benefit of the fibrous morphology. During mask wear, a bend radius in the range of centimeters can be expected. Using the equation above, the surface bending strain will be less than 0.02% during wear, thus negligibly impacting the respiratory signal.

Cyclical bending was performed to evaluate the mechanical stability of the ILG-on-PET and mask-integrated ILG devices, as shown in Supporting Information, Figure S17. In accordance with the dependence of current on the bend angle (Figure 6c), the current levels oscillate in response to cycling (Supporting Information, Figure S17b), with no signs of abrupt damage in response to angles up to 150° and 1000 cycles. The baseline gradually shifts toward lower currents, even in the absence of bend cycling, which we attribute to a charge buildup under DC bias conditions. Over the course of 1 h, the baseline lowers by 55% for the PET-based device and 25% for the mask-based device. A similar baseline drift was observed in previous ILG sensors operating under DC bias conditions.^28^ In practice, for respiratory sensing, baseline drift can be eliminated by taking the first derivative of the current signal or through other signal processing methods.^40^ The minor differences between the baseline and cycling data show that the printed electrodes and the ILG exhibit excellent mechanical resilience under repeated external stress, even at high bending angles.

When worn as a wearable respiratory sensor, the mask-integrated ILG device responds sensitively to normal breathing and apnea (stopped breathing; Figure 7a), rapid breathing (Figure 7b), and coughing (Figure 7c). The current increases during exhalation when humid air from the lungs reaches the sensor, and the current decreases during inhalation when the local humidity decreases. The distinct responses to inhalation and exhalation make it possible to extract the respiratory rate (RR). In the case of Figure 7b, the RR for normal breathing and rapid breathing are 18 bpm and 40 bpm, respectively. In the case of Figure 7c, the coughing signals appear sharper and taller than the normal breathing signals, showing the potential to extract health information from the measured respiratory signals. Data recorded from two users (one male and one female), breathing normally and with interspersed coughs or sighs, exhibit distinct individual differences (Figure 7d,e). It should be noted that the microenvironment inside a mask leads to humidity buildup, resulting in an average RH around 75–85%,^48–50^ which is within the high RH regime where the sensor has the highest sensitivity. During rapid breathing, the local humidity can even be higher than 90%.^49^ A transient baseline humidity level, as the mask’s microclimate evolves relative to the surrounding environment, can be a further source of baseline current drift during measurement, which can be accounted for through signal processing, as discussed earlier.

**Figure 7:**
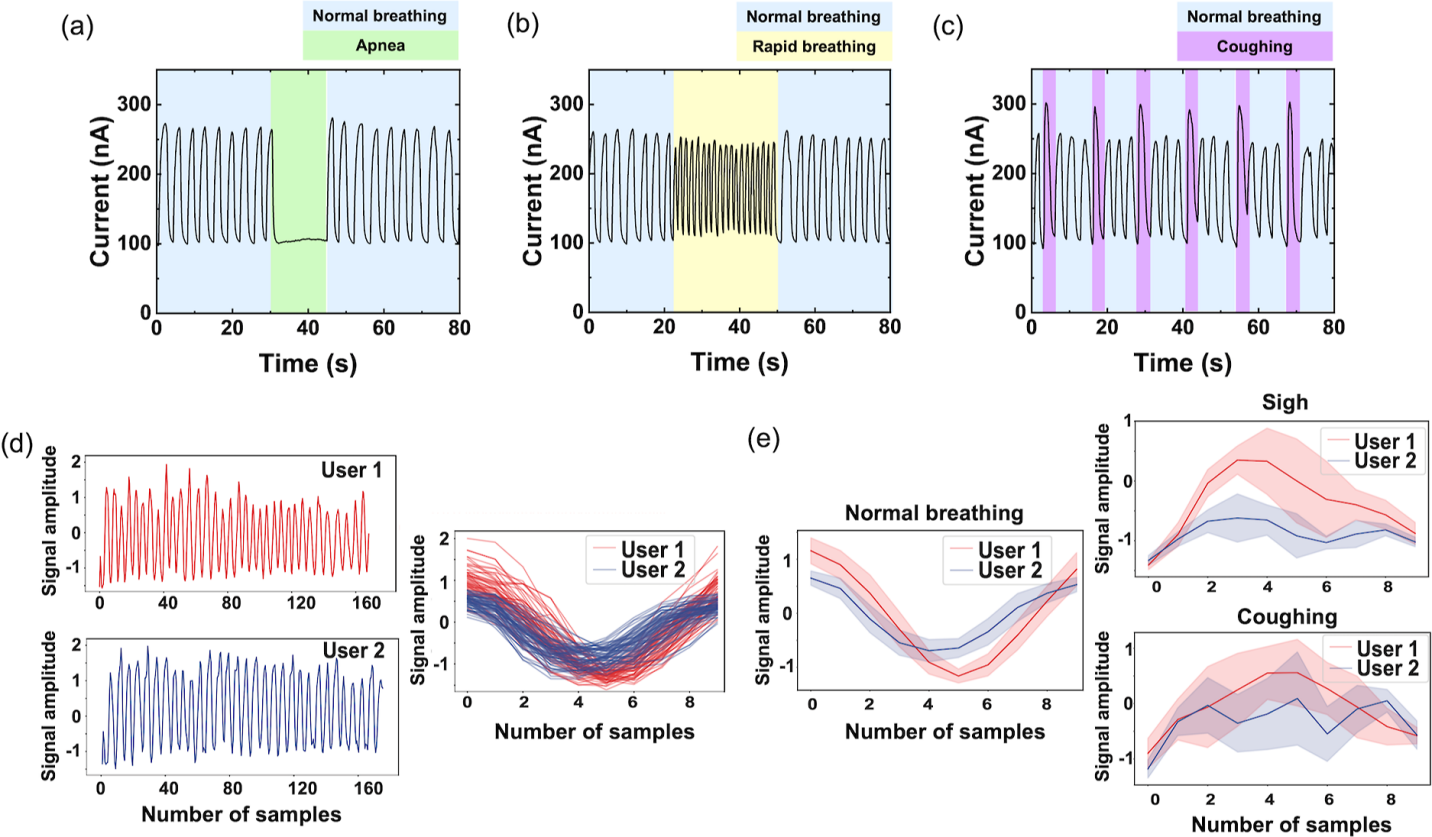
Mask-integrated ILG device responds to normal breathing and periods of (a) apnea, (b) rapid breathing, and (c) coughing. (d) Typical breathing cycles from two users. (e) Average signals for normal breathing, coughing, and sighing. The number of samples represents the time multiplied by the sampling rate.

AI analysis can play an important role in interpreting subtle changes in breathing patterns and classifying respiratory events such as coughing or rapid breathing. By integration of AI, the system can move beyond signal detection to symptom recognition, allowing for personalized health monitoring and early intervention. This is particularly valuable in remote or underserved areas, where access to clinical assessments is limited and automated respiratory tracking can enhance healthcare delivery. A summary of humidity-based respiratory sensor features reported in the literature is shown in Supporting Information, Table S1.

Two scenarios were tested to evaluate the system’s adaptability for new users: (1) a general model trained on one user and applied directly to another and (2) the same model adapted using a small amount of data from the new user. The first scenario targets the ideal usage scenario in which the AI analysis model is developed and trained based on a set of general population data; a new user would then directly use the sensing mask without any additional initialization. Unsurprisingly, this approach does not achieve ideal accuracy for classifying respiratory events, given the diversity of individual breathing characteristics (Figure 7e). To improve accuracy, the second scenario adopts transfer learning, which employs a small set of user data for user-specific calibration. This solution can significantly improve the performance, offering a practical compromise. Under the two outlined scenarios, we achieved accuracies of 81% and 91%, respectively, in detecting coughing events that were interspersed with regular breathing. The future need for transfer learning (user-specific adaptation) may be reduced by using larger multiuser training data sets, data augmentation (e.g., time warping), and more advanced AI models (e.g., domain adaptation). These preliminary experiments and analysis demonstrate the potential of AI-powered wearable sensors to monitor an individual’s respiratory patterns and recognize conditions that are of clinical significance.

## Conclusions

In summary, we showed that ILG-based humidity sensors can be scalably integrated onto microfibrous polypropylene face masks for wearable health monitoring. The sensors exhibit enhanced sensitivity at the high humidity levels that are present near the nose or mouth during exhalation. In addition, the sensor response and recovery times are similar to the time scale of breathing, enabling a distinct response to normal and rapid breathing.

The role of the ILG composition was systematically studied on planar substrates. It was found that the device sensitivity and response kinetics are inversely related, resulting from an increased hydrophilicity for a higher IL content. The increased hydrophilicity leads to greater amounts of water adsorption, greater proton generation following water dissociation, and, in turn, larger currents under an applied bias. On the other hand, a more extensive water layer takes longer to adsorb and desorb, resulting in slowed kinetics.

Mechanical testing of freestanding films shows that the ILG thin film can be elongated up to 120% strain while maintaining its humidity-sensing ability. In addition, ILG devices on microfibrous mask substrates were less sensitive to bending than devices on PET substrates; four times less sensitive under extreme bending at 150°. Interestingly the sign of the current change under bending was the opposite for mask-integrated and ILG-on-PET devices. This can be explained by the coating of the ILG around the mask’s microfibers, leading to simultaneous compression and tension during bending, whereas the planar devices were only subjected to tension. The compressed ILG results in a path of least resistance that is favored for proton flow.

Finally, we showed that the mask-integrated sensor can be coupled with AI analysis to enable cough detection with 91% accuracy. This study shows that the direct integration of active materials and printed electrodes onto microfibrous substrates is promising for the scalable manufacturing of health sensors. This approach preserves the porous nature of the substrate and leads to superior mechanical properties compared with conventional planar devices. In combination with wireless electronics for communication with a smartphone and AI-powered pattern classification, mask-integrated respiratory sensors hold promise for enhancing at-home patient monitoring, telemedicine, and treatment of under-resourced populations.

## Methods

### ILG Solution Preparation

The ILG was prepared in a nitrogen-filled glovebox by using a copolymer/IL/acetone weight ratio of 1:0.67:7, 1:1.5:7, and 1:4:7. These compositions are referred to as 40, 60, and 80 wt %, respectively, to reflect the weight percent of IL in the final ILG. The P­(VDF-HFP) copolymer was purchased from Sigma-Aldrich. The copolymer was first dissolved in acetone and stirred on a hot plate at 260 rpm and 50 °C for 2 h. The IL [EMIM]­[TFSI], purchased from TCI America, was then added to the solution and stirred at 260 rpm and 50 °C for 24 h.

### Sample and Device Preparation

Polyethylene terephthalate (PET) with an area of 2.5 cm × 2.0 cm was cleaned with Fisherbrand Sparkleen soap and water, sonicated sequentially in acetone, isopropanol, and deionized water for 5 min each, and then exposed to UV–ozone. Gold electrodes were thermally evaporated onto the PET by depositing 7 nm of chromium and 50 nm of gold through a shadow mask. The electrode separation was 80 μm, and the electrode width was 2.5 mm. For screen-printed devices, silver/silver chloride electrodes were printed onto PET or the melt-blown polypropylene filter layer of a three-layer disposable mask (Omey) using E2414 Ag/AgCl ink from Ercon Inc., with an electrode gap of 0.5 mm and an electrode width of 2.5 mm. The printed electrodes were then annealed at 120 °C for 5 min. ILG films on PET were prepared by spin-coating 50 °C ILG solution at 6000 rpm for 30 s and annealing at 70 °C for 24 h to remove residual solvents. The spin-coated film thicknesses were 1.7 μm, 2.8 μm, and 5.0 μm for the 40, 60, and 80 wt % ILG, respectively. Conformal ILG coating of mask substrates was achieved by drop-casting a small volume (10 μL) of dilute 50 °C, 60 wt % ILG solution (using four times the standard acetone amount), and annealing at 70 °C for 24 h. Free-standing ILG films (60 wt %) for testing under strain were prepared by peeling spin-coated and annealed ILG films off glass substrates. Thick (210 μm) free-standing ILG films (60 wt %) were prepared for bending tests by drop-casting 500 μL of 50 °C ILG solution into a 2.5 cm diameter vial, heating the films at 70 °C for 24 h, then peeling and cutting the films.

### Material and Device Characterization

FTIR spectra were recorded by using a Bruker ALPHA II FT-IR spectrometer. Surface topography was measured using an AIST-NT CombiScope atomic force microscope and BudgetSensors cantilevers with a force constant of 40 N/m. Water contact angles were measured using a Theta Lite optical tensiometer (Biolin Scientific) and deionized water. Scanning electron microscopy (SEM) (Zeiss Supra 55VP) and energy-dispersive spectroscopy (EDS) (EDAX Genesis) were performed using a 15 keV beam energy on carbon-coated ILG samples. Device sensitivity was measured by using a Keithley 2636A SourceMeter instrument to monitor the current under an applied voltage bias of 2 V, while the humidity was varied in a humidity control chamber. To analyze the response and recovery time, devices were quickly moved between closed flasks containing a saturated solution of lithium chloride (Sigma-Aldrich) and sodium chloride (Fisher Scientific) that created atmospheres with 11% RH and 75% RH, respectively.^5,7^ Stress–strain curves and performance under bending and stretching were recorded by using a Mark-10 ESM 303 test stand. For devices under strain, the humidity was varied by using a linear translation stage to move a blocker between the device and the humidity source.

### Artificial Intelligence Analysis

In total, 158 and 58 breathing cycles were collected from Subjects 1 and 2, respectively, among which there were 98 normal breathing samples, 46 coughing samples, and 38 sighing samples. A fourth-order Butterworth filter was applied to the raw data, followed by a detrending technique to remove low-frequency transients. Continuous wavelet transform and zero-crossing rate methods were then adopted to detect and segment each breathing cycle. For our analysis, we extracted a set of statistical and morphological features of the time-series data and adopted a standard support vector machine.

## Supplementary Material



## Notes

ILG film characterization; mask-integrated ILG device; and mechanical testing (PDF)

## References

[1] Mortality in the United States, 2022; National Center for Health Statistics: Hyattsville, MD, 2024. 41284807

[2] Tan W. C., Ng T. P. (2008). COPD in
Asia: where East meets West. Chest.

[3] Siafakas N. M., Vermeire P., Pride N. B., Paoletti P., Gibson J., Howard P., Yernault J. C., Decramer M., Higenbottam T., Postma D. S. (1995). Optimal
assessment and management of chronic
obstructive pulmonary disease (COPD). The European Respiratory Society
Task Force. Eur. Respir. J..

[4] Larsson K., Janson C., Ställberg B., Lisspers K., Olsson P., Kostikas K., Gruenberger J.-B., Gutzwiller F. S., Uhde M., Jorgensen L. (2019). Impact of COPD diagnosis
timing on clinical and economic outcomes: the ARCTIC observational
cohort study. Int. J. Chronic Obstruct. Pulm.
Dis..

[5] Liu X., Zhang D., Wang D., Li T., Song X., Kang Z. (2021). A humidity sensing and respiratory
monitoring system constructed
from quartz crystal microbalance sensors based on a chitosan/polypyrrole
composite film. J. Mater. Chem. A.

[6] Shin J., Jeong B., Kim J., Nam V. B., Yoon Y., Jung J., Hong S., Lee H., Eom H., Yeo J. (2020). Sensitive Wearable Temperature
Sensor with Seamless
Monolithic Integration. Adv. Mater..

[7] Ma L., Wu R., Patil A., Zhu S., Meng Z., Meng H., Hou C., Zhang Y., Liu Q., Yu R. (2019). Full-Textile
Wireless Flexible Humidity Sensor for Human Physiological Monitoring. Adv. Funct. Mater..

[8] Liu Q., Tai H., Yuan Z., Zhou Y., Su Y., Jiang Y. (2019). A High-Performances
Flexible Temperature Sensor Composed of Polyethyleneimine/Reduced
Graphene Oxide Bilayer for Real-Time Monitoring. Adv. Mater. Technol..

[9] Tao L. Q., Zhang K. N., Tian H., Liu Y., Wang D. Y., Chen Y. Q., Yang Y., Ren T. L. (2017). Graphene-Paper
Pressure
Sensor for Detecting Human Motions. ACS Nano.

[10] Li M., Li H., Zhong W., Zhao Q., Wang D. (2014). Stretchable conductive
polypyrrole/polyurethane (PPy/PU) strain sensor with netlike microcracks
for human breath detection. ACS Appl. Mater.
Interfaces.

[11] Vicente B.
A., Sebastião R., Sencadas V. (2024). Wearable Devices for Respiratory
Monitoring. Adv. Funct. Mater..

[12] Shen S., Zhou Q., Chen G., Fang Y., Kurilova O., Liu Z., Li S., Chen J. (2024). Advances in
wearable respiration
sensors. Mater. Today.

[13] Thacharodi A., Singh P., Meenatchi R., Tawfeeq Ahmed Z. H., Kumar R. R. S., V N., Kavish S., Maqbool M., Hassan S. (2024). Revolutionizing healthcare and medicine: The impact
of modern technologies for a healthier future—A comprehensive
review. Healthcare Sci..

[14] Mogera U., Sagade A. A., George S. J., Kulkarni G. U. (2014). Ultrafast response
humidity sensor using supramolecular nanofibre and its application
in monitoring breath humidity and flow. Sci.
Rep..

[15] Ferrus L., Guenard H., Vardon G., Varene P. (1980). Respiratory water loss. Respir.
Physiol..

[16] Mansour E., Vishinkin R., Rihet S., Saliba W., Fish F., Sarfati P., Haick H. (2020). Measurement of temperature and relative
humidity in exhaled breath. Sens. Actuators,
B.

[17] Borini S., White R., Wei D., Astley M., Haque S., Spigone E., Harris N., Kivioja J., Ryhänen T. (2013). Ultrafast
Graphene Oxide Humidity Sensors. ACS Nano.

[18] Wu J., Sun Y.-M., Wu Z., Li X., Wang N., Tao K., Wang G. P. (2019). Carbon Nanocoil-Based
Fast-Response and Flexible Humidity
Sensor for Multifunctional Applications. ACS
Appl. Mater. Interfaces.

[19] Zhu P., Ou H., Kuang Y., Hao L., Diao J., Chen G. (2020). Cellulose
Nanofiber/Carbon Nanotube Dual Network-Enabled Humidity Sensor with
High Sensitivity and Durability. ACS Appl. Mater.
Interfaces.

[20] Wang J., Li L., Chen G., Wang C. (2021). Enhanced Humidity Sensing Performance
of the La2Ti2O7/La­(OH)­3 Nanocomposite. ACS Appl.
Electron. Mater..

[21] Yang J., Shi R., Lou Z., Chai R., Jiang K., Shen G. (2019). Flexible Smart
Noncontact Control Systems with Ultrasensitive Humidity Sensors. Small.

[22] Duan Z., Jiang Y., Yan M., Wang S., Yuan Z., Zhao Q., Sun P., Xie G., Du X., Tai H. (2019). Facile, Flexible, Cost-Saving, and
Environment-Friendly Paper-Based
Humidity Sensor for Multifunctional Applications. ACS Appl. Mater. Interfaces.

[23] Dai J., Zhao H., Lin X., Liu S., Liu Y., Liu X., Fei T., Zhang T. (2019). Ultrafast Response Polyelectrolyte
Humidity Sensor for Respiration Monitoring. ACS Appl. Mater. Interfaces.

[24] Yang Y., Wang J., Lou J., Yao H., Zhao C. (2023). Fast response
humidity sensor based on hyperbranched zwitterionic polymer for respiratory
monitoring and non-contact human machine interface. Chem. Eng. J..

[25] Jansen J. C., Friess K., Clarizia G., Schauer J., Izák P. (2011). High Ionic
Liquid Content Polymeric Gel Membranes: Preparation and Performance. Macromolecules.

[26] Yao X., Zhang S., Qian L., Wei N., Nica V., Coseri S., Han F. (2022). Super Stretchable, Self-Healing,
Adhesive Ionic Conductive Hydrogels Based on Tailor-Made Ionic Liquid
for High-Performance Strain Sensors. Adv. Funct.
Mater..

[27] Jin M. L., Park S., Kim J.-S., Kwon S. H., Zhang S., Yoo M. S., Jang S., Koh H.-J., Cho S.-Y., Kim S. Y. (2018). An
Ultrastable Ionic Chemiresistor Skin with an Intrinsically
Stretchable Polymer Electrolyte. Adv. Mater..

[28] Le
Bideau J., Viau L., Vioux A. (2011). Ionogels, ionic liquid
based hybrid materials. Chem. Soc. Rev..

[29] Wang H., Wang Z., Yang J., Xu C., Zhang Q., Peng Z. (2018). Ionic Gels and Their Applications
in Stretchable Electronics. Macromol. Rapid
Commun..

[30] Seddon K. R. (2003). A taste
of the future. Nat. Mater..

[31] Armand M., Endres F., MacFarlane D. R., Ohno H., Scrosati B. (2009). Ionic-liquid
materials for the electrochemical challenges of the future. Nat. Mater..

[32] Ueki T., Watanabe M. (2008). Macromolecules in Ionic Liquids: Progress, Challenges,
and Opportunities. Macromolecules.

[33] Park S.-J., Jeon J.-Y., Ha T.-J. (2022). Wearable
humidity sensors based on
bar-printed poly­(ionic liquid) for real-time humidity monitoring systems. Sens. Actuators, B.

[34] Zhou Y., Zhao L., Jia Q., Wang T., Sun P., Liu F., Yan X., Wang C., Sun Y., Lu G. (2022). Multifunctional
Flexible Ionic Skin with Dual-Modal Output Based on Fibrous Structure. ACS Appl. Mater. Interfaces.

[35] Zhao X., Zhou K., Zhong Y., Liu P., Li Z., Pan J., Long Y., Huang M., Brakat A., Zhu H. (2021). Hydrophobic
ionic liquid-in-polymer composites for ultrafast, linear response
and highly sensitive humidity sensing. Nano
Res..

[36] Xiao S., Nie J., Tan R., Duan X., Ma J., Li Q., Wang T. (2019). Fast-response
ionogel humidity sensor for real-time monitoring of
breathing rate. Mater. Chem. Front..

[37] Yang Y., Lou J., Qi D., Zhao C. (2024). Flexible and transparent humidity
sensors based on hyperbranched poly­(ionic liquid)­s for wearable sensing. Sens. Actuators, B.

[38] Zhang K., Li Z., Zhang J., Zhao D., Pi Y., Shi Y., Wang R., Chen P., Li C., Chen G. (2022). Biodegradable
Smart Face Masks for Machine Learning-Assisted Chronic
Respiratory Disease Diagnosis. ACS Sens..

[39] Zhong J., Li Z., Takakuwa M., Inoue D., Hashizume D., Jiang Z., Shi Y., Ou L., Nayeem M. O. G., Umezu S. (2022). Smart Face Mask Based
on an Ultrathin Pressure
Sensor for Wireless Monitoring of Breath Conditions. Adv. Mater..

[40] Guder F., Ainla A., Redston J., Mosadegh B., Glavan A., Martin T. J., Whitesides G. M. (2016). Paper-Based
Electrical Respiration
Sensor. Angew. Chem. Int. Ed..

[41] Wu G., Du H., Lee D., Cha Y. L., Kim W., Zhang X., Kim D.-J. (2022). Polyaniline/Graphene-Functionalized
Flexible Waste
Mask Sensors for Ammonia and Volatile Sulfur Compound Monitoring. ACS Appl. Mater. Interfaces.

[42] Aoki K., Sugawara-Narutaki A., Doi Y., Takahashi R. (2022). Structure
and Rheology of Poly­(vinylidene difluoride-co-hexafluoropropylene)
in an Ionic Liquid: The Solvent Behaves as a Weak Cross-Linker through
Ion–Dipole Interaction. Macromolecules.

[43] Gao L., Jiang W., Zhang X., Sun Y., Chen K., Li W., Xie H., Liu J. (2024). A self-healing
poly­(ionic liquid)
block copolymer electrolyte enabled by synergetic dual ion-dipole
interactions. Chem. Eng. J..

[44] Hill B., Annesley S. H. (2020). Monitoring respiratory rate in adults. Br. J. Nurs..

[45] Hunt P. A., Ashworth C. R., Matthews R. P. (2015). Hydrogen bonding in ionic liquids. Chem. Soc. Rev..

[46] Wrobel P., Kubisiak P., Eilmes A. (2022). Hydrogen Bonding and Infrared Spectra
of Ethyl-3-methylimidazolium Bis­(trifluoromethylsulfonyl)­imide/Water
Mixtures: A View from Molecular Dynamics Simulations. J. Phys. Chem. B.

[47] White M. S., Kaltenbrunner M., Głowacki E. D., Gutnichenko K., Kettlgruber G., Graz I., Aazou S., Ulbricht C., Egbe D. A. M., Miron M. C. (2013). Ultrathin,
highly flexible
and stretchable PLEDs. Nat. Photonics.

[48] Cherrie J. W., Wang S., Mueller W., Wendelboe-Nelson C., Loh M. (2019). In-mask temperature and humidity
can validate respirator wear-time
and indicate lung health status. J. Exposure
Sci. Environ. Epidemiol..

[49] Fois, A. ; Tocco, F. ; Dell’Osa, A. ; Melis, L. ; Bertelli, U. ; Concu, A. ; Manuello Bertetto, A. ; Serra, C. Innovative Smart Face Mask to Protect Workers from COVID-19 Infection. 2021 IEEE International Symposium on Medical Measurements and Applications (MeMeA); IEEE, 2021; pp 1–6.

[50] Roberge R. J., Kim J.-H., Benson S. (2012). N95 Filtering Facepiece Respirator
Deadspace Temperature and Humidity. J. Occup.
Environ. Hyg..

