# Supplementary material for: Direct Integration of Ionic Liquid Gel Sensors onto Microfibrous Face Mask Substrates for Wearable Respiratory Health Monitoring
Source: ACS Appl Bio Mater. 2026 Feb 17;9(6):2866–75. doi: 10.1021/acsabm.5c01939 (PMC12997150; doi:10.1021/acsabm.5c01939)
Supplement: Supplementary file 1 [file mt5c01939_si_001.pdf]

# Supporting Information

# Direct Integration of Ionic Liquid Gel Sensors onto Microfibrous Face Mask Substrates for Wearable Respiratory Health Monitoring

*Ziqi Qing,*[a] *Seokmin Choi,*[b] *Matthew S. Brown,*[c] *Md Abid Hasan Shanto,*[a] *Yincheng Jin,*[d] *Ahyeon Koh,*[c] *Zhanpeng Jin,*[b] *Jeffrey M. Mativetsky*[a, e]*

[a] Materials Science and Engineering, Binghamton University, Binghamton, New York 13902, United States

[b] Department of Computer Science and Engineering, University at Buffalo, Buffalo, New York 14260, United States

[c] Department of Biomedical Engineering, Binghamton University, Binghamton, New York 13902, United States

[d] School of Computing, Binghamton University, Binghamton, New York 13902, United States

[e] Department of Physics, Applied Physics and Astronomy, Binghamton University, Binghamton, New York 13902, United States

* Corresponding Author: jmativet@binghamton.edu

1. ILG Film Characterization ........................................ 1
2. PET-Based ILG Device ........................................ 3
3. Mask-Integrated ILG Device ........................................ 6
4. Mechanical Testing ........................................ 9
5. Literature comparison ........................................ 11
6. References ........................................ 12

## 1. ILG Film Characterization

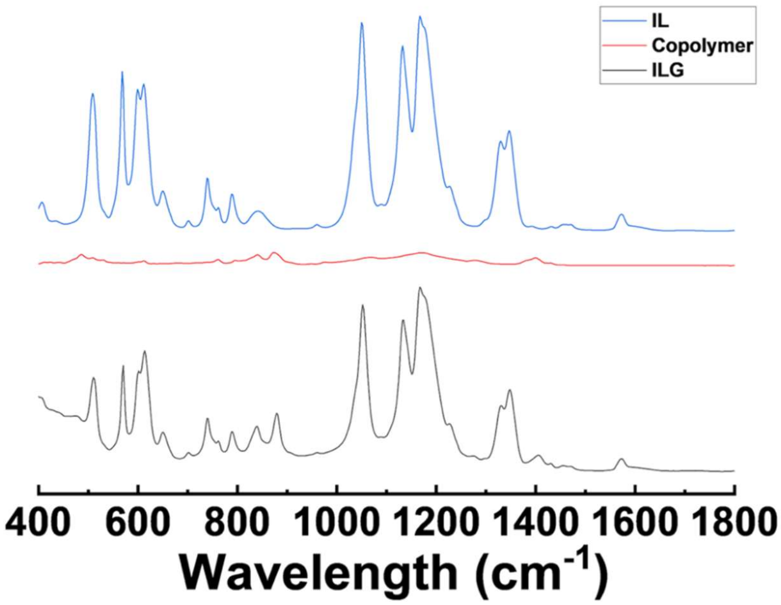


**Figure S1.** FTIR spectra of IL, copolymer, and the ILG.

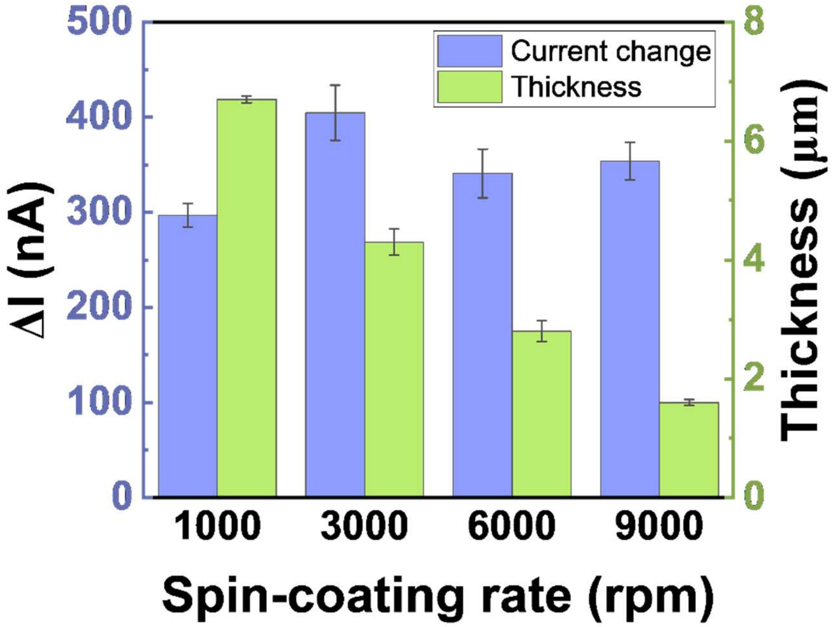


**Figure S2.** Film thickness and current change 200 s after switching from 11% to 75% RH for 60 wt. % ILG spin-coated at 1000 rpm, 3000 rpm, 6000 rpm, and 9000 rpm.

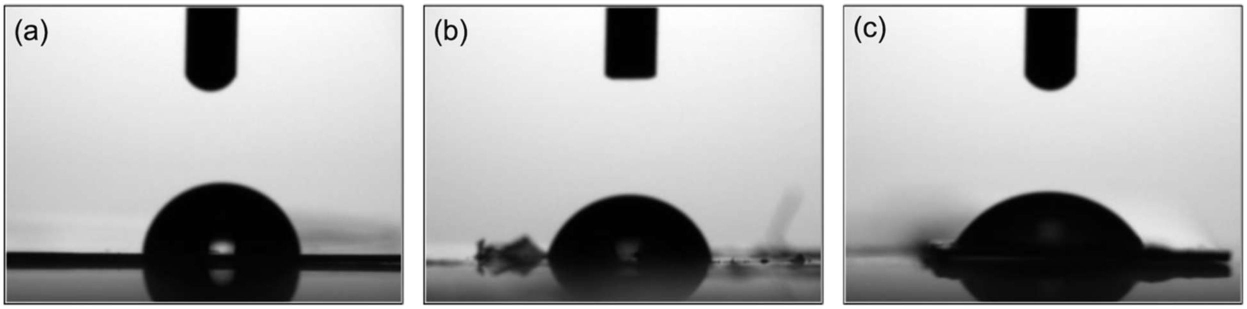


**Figure S3.** Water contact angle for (a) 40 wt. % ILG (b) 60 wt. % ILG, and (c) 80 wt. % ILG.

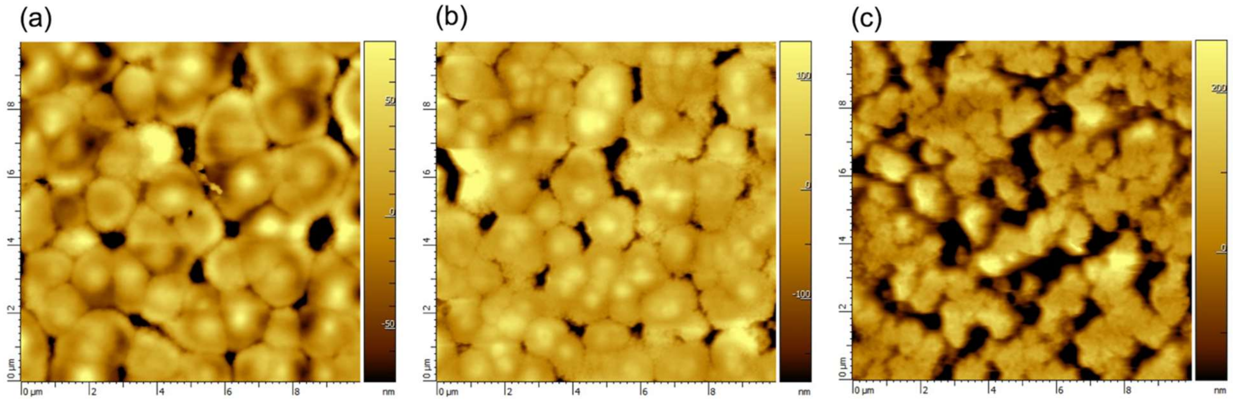


**Figure S4.** AFM topography for (a) 40 wt. % ILG, (b) 60 wt. % ILG, and (c) 80 wt. %ILG.

## 2. PET-Based ILG Device

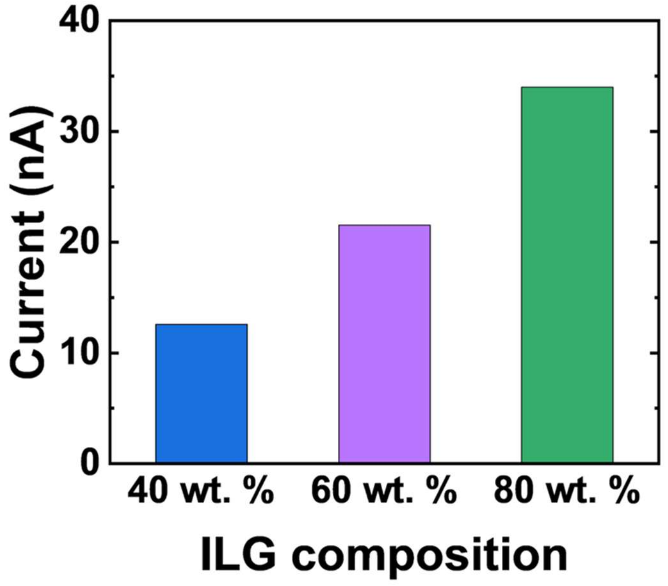


**Figure S5.** Ionic current at 0% RH for all three ILG compositions.

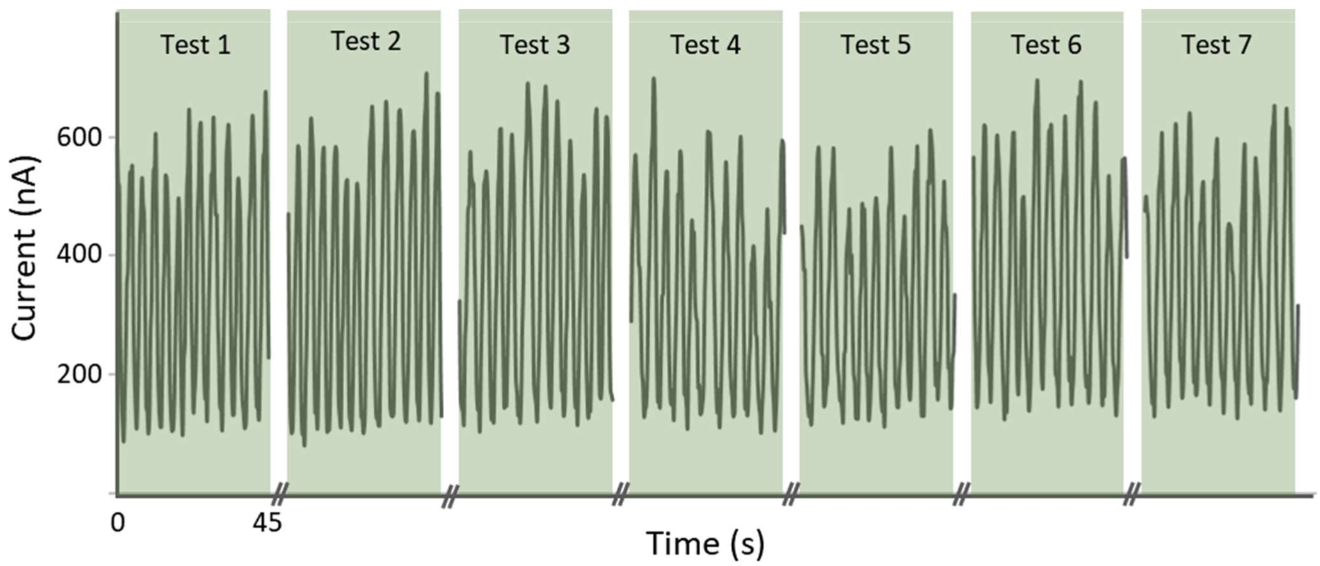


**Figure S6**. Current response to seven breathing tests spread over a 24-hour period showing no signs of degradation.

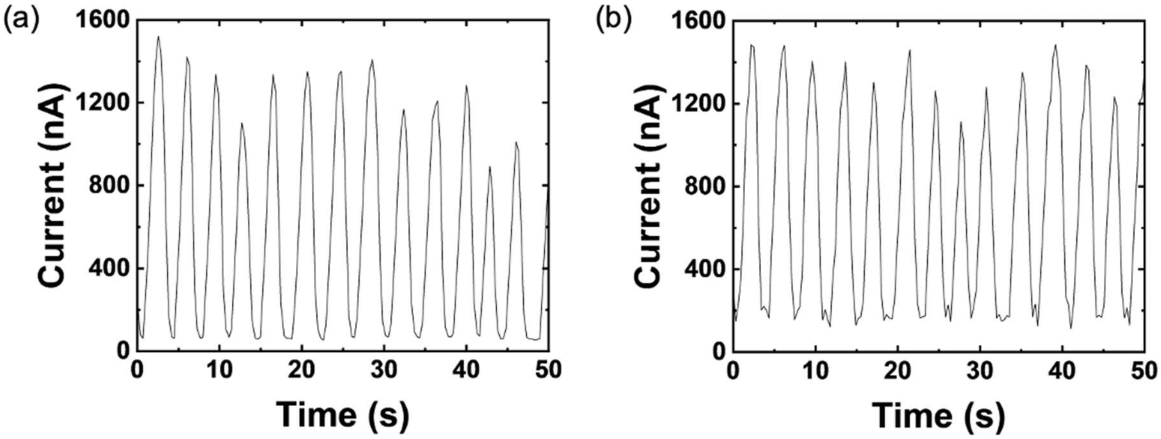


**Figure S7.** Sensing performance of the ILG film to breathing (a) before and (b) after the storage in nitrogen for 4 months.

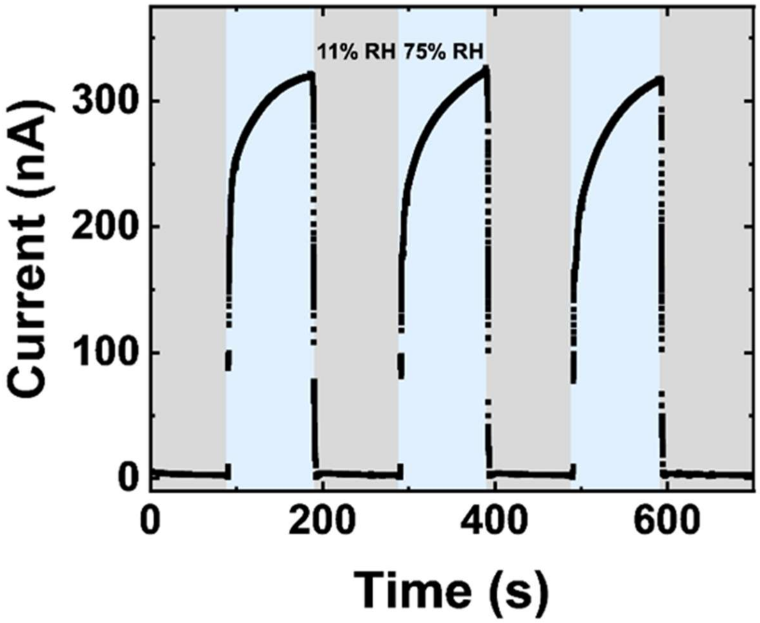


**Figure S8**. Repeatable current signals during rapid switching between 11% and 75% RH.

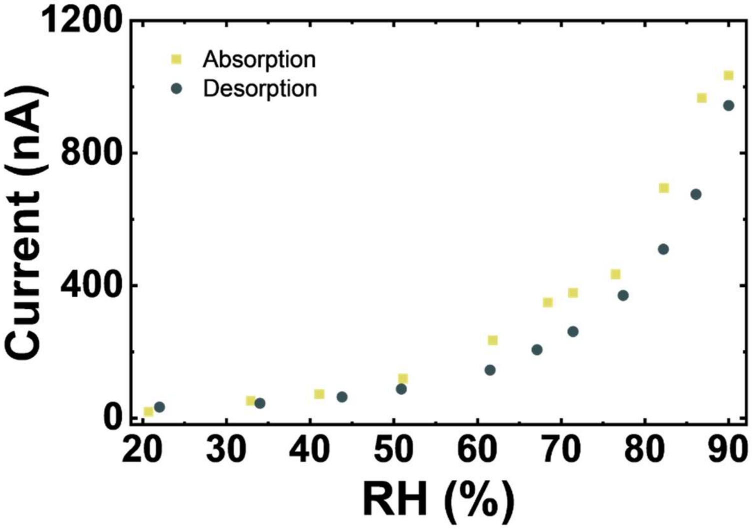


**Figure S9.** Absorption and desorption curves for 60 wt. % ILG, showing a 6.6% hysteresis error.

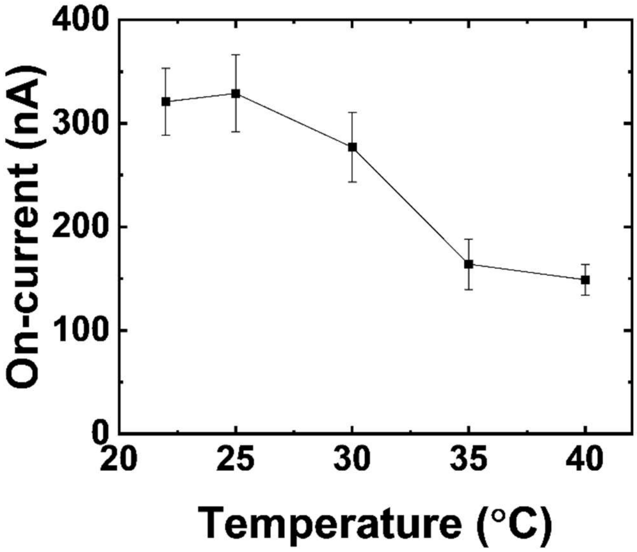


**Figure S10**. Average peak current (on-current) during breathing, as a function of ILG sensor temperature.

## 3. Mask-Integrated ILG Device

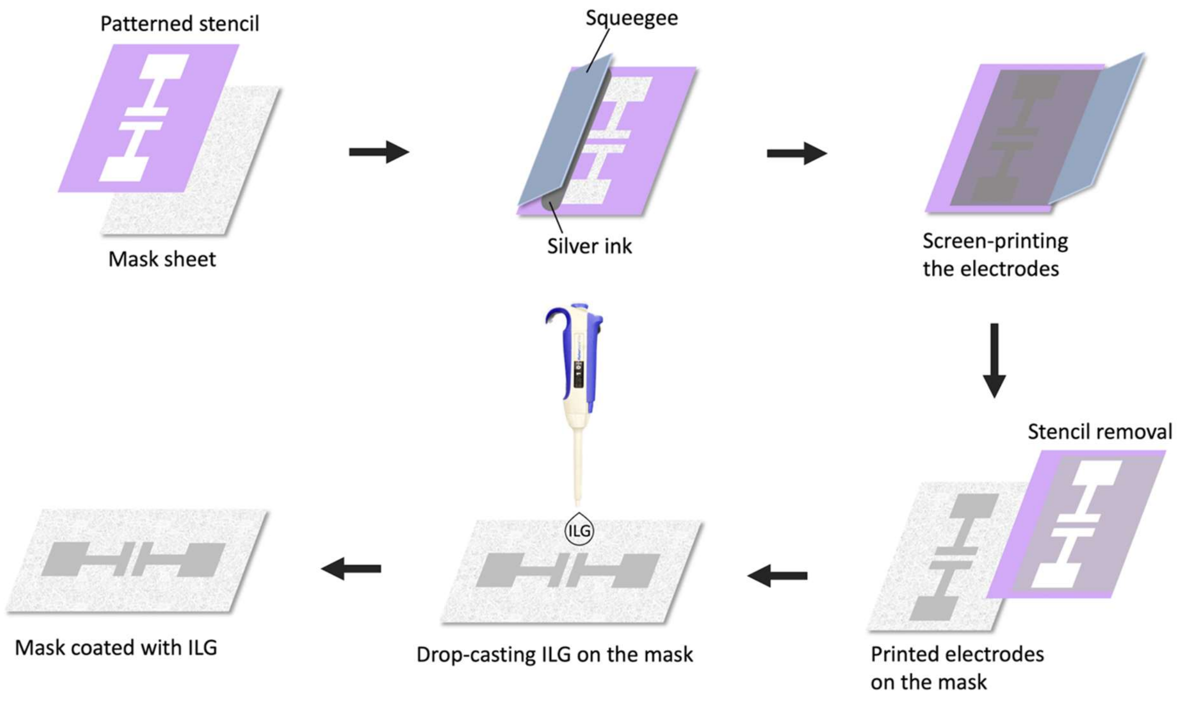


**Figure S11.** Fabrication steps for mask-integrated ILG device.

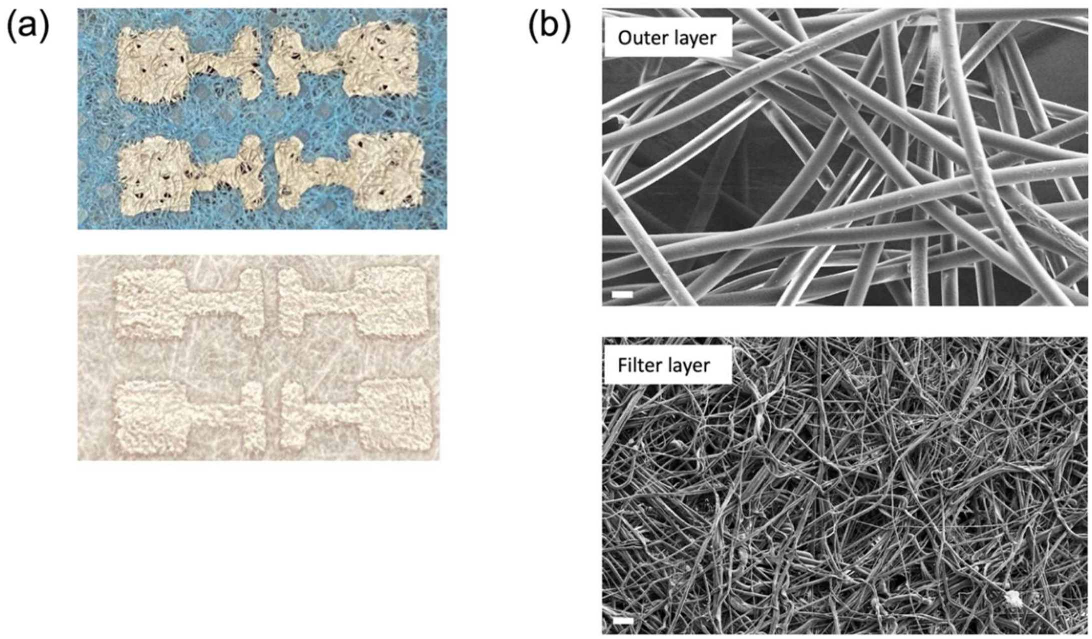


**Figure S12.** (a) Silver electrodes printed on the face mask's outer layer (top) and filter layer (bottom). (b) SEM images of a pristine mask’s outer layer and filter layer. The scale bars represent 30 $\mu m$.

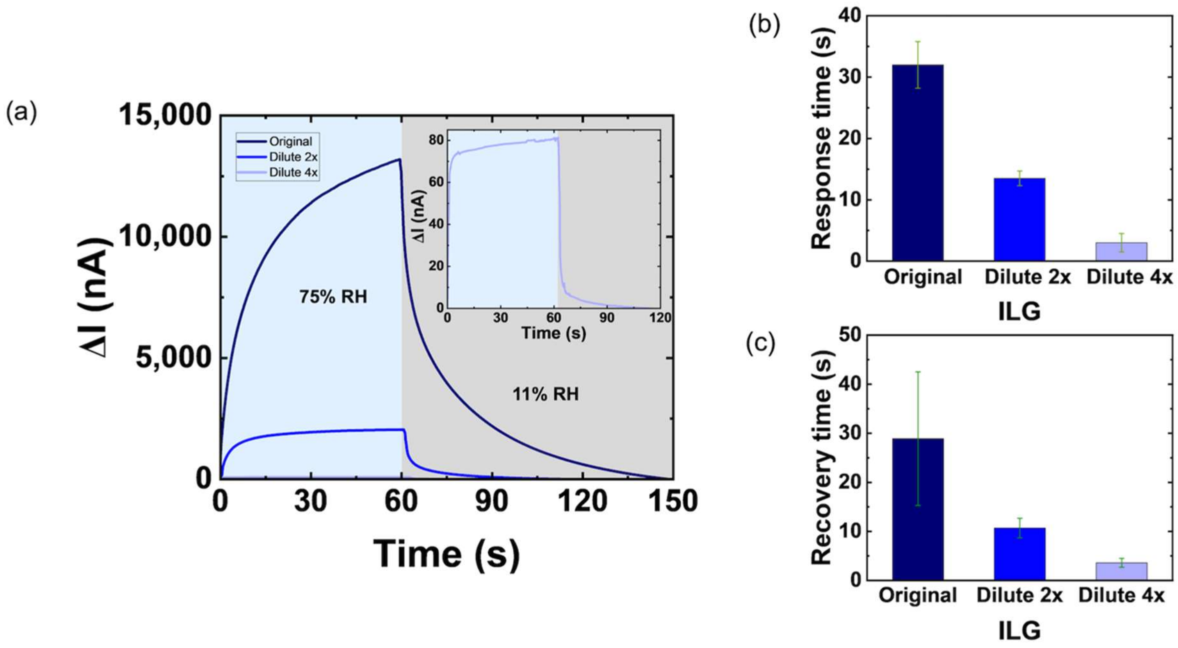


**Figure S13.** (a) Current change during rapid switching between 11% and 75% RH for ILG devices using the original ILG solution, the ILG solution diluted by a factor of two (two times the acetone wt. %), and the ILG solution diluted by a factor of four (four times the acetone wt. %). The inset shows an expanded view of the current change for the 4x diluted ILG. (b) Response time and (c) recovery time for the three dilution levels.

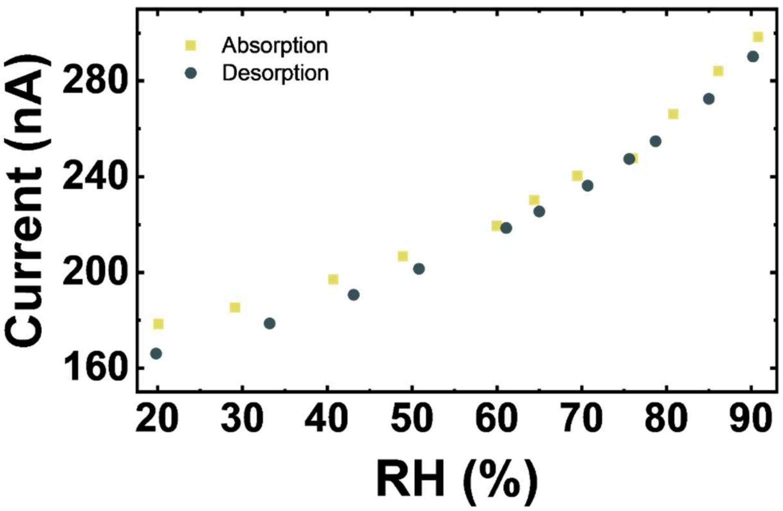


**Figure S14.** Absorption and desorption curves for a mask-integrated ILG device (60 wt. % ILG), showing a 3.0% hysteresis error.

## 4. Mechanical Testing

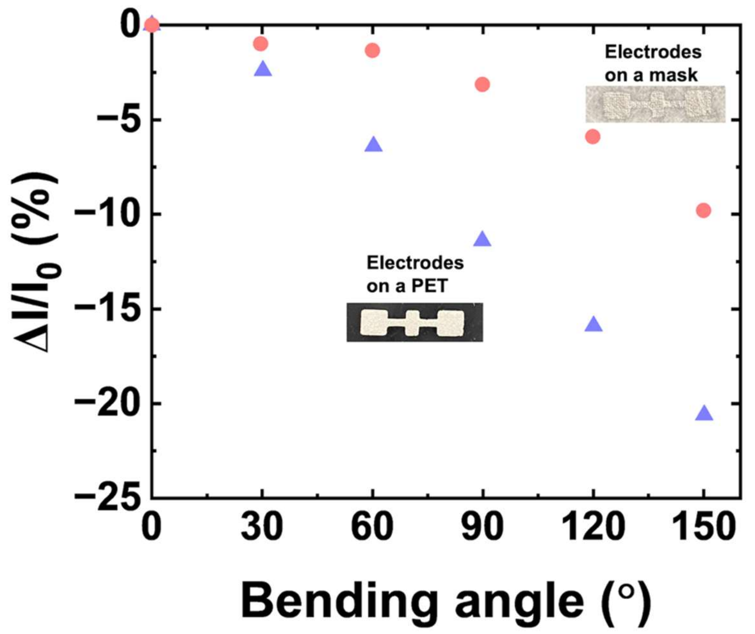


**Figure S15.** Percent current change as a function of the bending angle for continuous screen-printed electrodes on PET and a mask substrate.

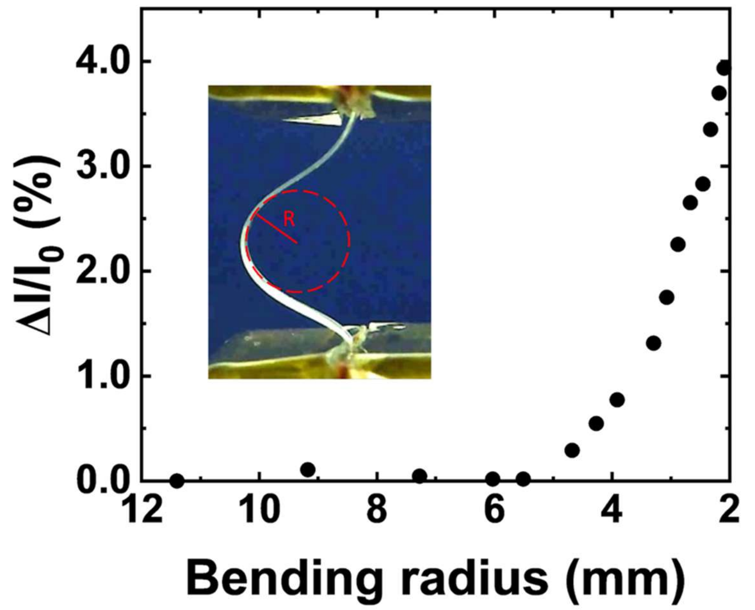


**Figure S16.** Percent current change for a free-standing ILG as a function of bending radius. The inset shows the defined bending radius. The ambient condition was 33% RH.

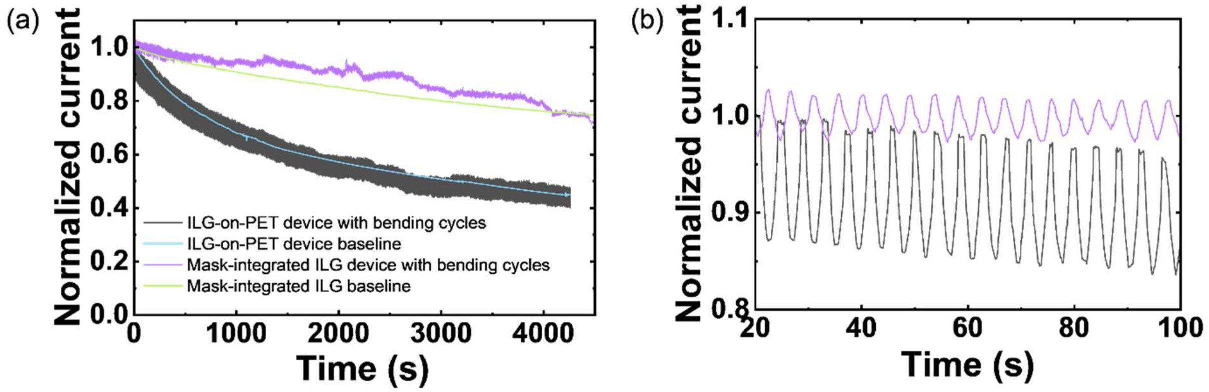


**Figure S17. (a)** The current change of the ILG-on-PET device and the mask-integrated ILG device during 1000 bending cycles with a bending angle of 150° over the course of an hour. Two baseline curves are included to show the current decrease without bend cycling. (b) The expanded view of the current patterns of the two devices during the early cycles.

## 5. Literature comparison

**Table S1.** Comparison of humidity sensors.

| Reference | Active sensing materials | Sensor integration | AI functionality |
|---|---|---|---|
| 1 | ILG fibers membrane ([EMIM][TFSI] and TPU) | Sensor attached inside mask | No |
| 2 | ILG fibers membrane ([EMIM][TFSI] and P(VDF-HFP)) | No | No |
| 3 | Cellulose paper | Sensor attached inside mask | No |
| 4 | CS/PPy composite film | Sensor attached inside mask | No |
| 5 | Functional yarns | Sensor stitched inside mask | No |
| 6 | PVDF/POS-Prs-TFSI | Sensor attached inside mask | No |
| 7 | Silicon nanoparticle | Sensor attached between mask layers | No |
| 8 | Acid-doped graphdiyne oxide | Sensor attached between mask layers | No |
| 9 | PEDOT:PSS | Sensor attached inside mask | No |
| 10 | ILG ([EMIM][TFSI] and PVDF-HFP) | Sensor attached inside mask | Mouth vs nose breathing |
| 11 | AuNPs/PAH composite | Sensor attached inside mask | Speech recognition |
| 12 | PVDF/TPU/C membrane | Sensor attached inside mask | Airflow control |
| 13 | Polyimide nanoforests | Sensor attached to mask exterior | Behavior (e.g., walking, sleep, apnea) detection |
| 14 | Ag/PEDOT | Sensor attached inside mask | User identification |
| This work | ILG ([EMIM][TFSI] and P(VDF-HFP)) | Sensor integrated with mask layer | Cough detection |

## 6. References


(1) Zhou, Y.; Zhao, L.; Jia, Q.; Wang, T.; Sun, P.; Liu, F.; Yan, X.; Wang, C.; Sun, Y.; Lu, G. Multifunctional Flexible Ionic Skin with Dual-Modal Output Based on Fibrous Structure. *ACS Applied Materials & Interfaces* **2022**, *14* (49), 55109-55118.

(2) Zhao, X.; Zhou, K.; Zhong, Y.; Liu, P.; Li, Z.; Pan, J.; Long, Y.; Huang, M.; Brakat, A.; Zhu, H. Hydrophobic ionic liquid-in-polymer composites for ultrafast, linear response and highly sensitive humidity sensing. *Nano Research* **2021**, *14* (4), 1202-1209.

(3) Guder, F.; Ainla, A.; Redston, J.; Mosadegh, B.; Glavan, A.; Martin, T. J.; Whitesides, G. M. Paper-Based Electrical Respiration Sensor. *Angew Chem Int Ed Engl* **2016**, *55* (19), 5727-5732.

(4) Liu, X.; Zhang, D.; Wang, D.; Li, T.; Song, X.; Kang, Z. A humidity sensing and respiratory monitoring system constructed from quartz crystal microbalance sensors based on a chitosan/polypyrrole composite film. *Journal of Materials Chemistry A* **2021**, *9* (25), 14524-14533.

(5) Ma, L.; Wu, R.; Patil, A.; Zhu, S.; Meng, Z.; Meng, H.; Hou, C.; Zhang, Y.; Liu, Q.; Yu, R.; et al. Full-Textile Wireless Flexible Humidity Sensor for Human Physiological Monitoring. *Advanced Functional Materials* **2019**, *29* (43), 1904549.

(6) Lou, J.; Yang, Y.; Zhao, C. Flexible humidity sensors utilizing POSS-based amphoteric ionic liquids for multifunctional wearable bio-health sensing. *Chemical Engineering Journal* **2025**, *521*, 167077.

(7) Qin, J.; Zang, R.; Zhang, B.; Hu, X.; Yu, J.; Zhang, X.; Cheng, Y.; Zhang, X. Flexible, Self-Powered Humidity Sensor Based on Silicon Nanoparticle Composites for Moisture Monitoring in Wearables. *ACS Applied Nano Materials* **2025**, *8* (30), 15050-15059.

(8) Xue, Y.; Zhao, X.; Wu, F.; Hou, L.; Yu, P.; Li, L.; Mao, L. Near-Field Electrochemistry Enables a Wearable Sensor-Embedded Smart Facemask for Personalized Respiratory Assessment. *ACS Sensors* **2025**, *10* (3), 2378-2385.

(9) Beniwal, A.; Khandelwal, G.; Mukherjee, R.; Mulvihill, D. M.; Li, C. Eco-Friendly Textile-Based Wearable Humidity Sensor with Multinode Wireless Connectivity for Healthcare Applications. *ACS Applied Bio Materials* **2024**, *7* (7), 4772-4784.

(10) Zhang, Z.; Li, J.; Chen, H.; Wang, H.; Luo, Y.; Si, R.; Xie, R.; Tao, K.; Yang, B.-R.; Zhang, D.; et al. Scalable Fabrication of Uniform Fast-Response Humidity Field Sensing Array for Respiration Recognition and Contactless Human-Machine Interaction. *Advanced Functional Materials* **2025**, *35* (41), 2502583.

(11) Wang, J.; Zhang, H.; Wu, X.; Gao, M.; Wen, H.; Zhang, Z.; Makasheva, K.; Li, W. J.; Wang, Z. A Wearable AI-Driven Mask with Humidity-Sensing Respiratory Microphone for Non-Vocal Communication. *Advanced Science* **2025**, *12* (33), e04343.

(12) Wang, Y.; Qi, X.; Chen, L.; Cheng, Y.; Mu, Z.; Gu, X.; Li, S.; Song, Y.; He, X.; Huang, S. Transfer Learning-Assisted Porous Polymer Humidity Sensor for Powered Air-Purifying Mask. *Advanced Intelligent Systems* **2025**, *7* (4), 2400537.

(13) Yang, H.; Guo, Q.; Chen, G.; Zhao, Y.; Shi, M.; Zhou, N.; Huang, C.; Mao, H. An intelligent humidity sensing system for human behavior recognition. *Microsystems & Nanoengineering* **2025**, *11* (1), 17.

(14) Qiu, Y.; Li, L.; Xu, H.; Yang, R.; Song, Y.; Geng, Y.; Zhang, S.; Wang, X. High-Performance Humidity Sensor for Advanced Respiratory Monitoring and Educational Applications: Flexible and Wearable Design Based on Ag/PEDOT:PSS Binary Nanocomposites. *ACS Applied Electronic Materials* **2025**, *7* (7), 2776-2784.
